# Parkinson's Disease Phenotypes in Patient Neuronal Cultures and Brain Organoids Improved by 2‐Hydroxypropyl‐β‐Cyclodextrin Treatment

**DOI:** 10.1002/mds.28810

**Published:** 2021-10-12

**Authors:** Javier Jarazo, Kyriaki Barmpa, Jennifer Modamio, Cláudia Saraiva, Sònia Sabaté‐Soler, Isabel Rosety, Anne Griesbeck, Florian Skwirblies, Gaia Zaffaroni, Lisa M. Smits, Jihui Su, Jonathan Arias‐Fuenzalida, Jonas Walter, Gemma Gomez‐Giro, Anna S. Monzel, Xiaobing Qing, Armelle Vitali, Gerald Cruciani, Ibrahim Boussaad, Francesco Brunelli, Christian Jäger, Aleksandar Rakovic, Wen Li, Lin Yuan, Emanuel Berger, Giuseppe Arena, Silvia Bolognin, Ronny Schmidt, Christoph Schröder, Paul M.A. Antony, Christine Klein, Rejko Krüger, Philip Seibler, Jens C. Schwamborn

**Affiliations:** ^1^ Developmental and Cellular Biology Luxembourg Centre for Systems Biomedicine University of Luxembourg Esch‐sur‐Alzette Luxembourg; ^2^ OrganoTherapeutics société à responsabilité limitée simplifiée (SARL‐S) Esch‐sur‐Alzette Luxembourg; ^3^ Sciomics GmbH Heidelberg Germany; ^4^ Institute for Globally Distributed Open Research and Education Gothenburg Sweden; ^5^ Institute of Health Sciences China Medical University Shenyang China; ^6^ Translational Neuroscience, Luxembourg Centre for Systems Biomedicine University of Luxembourg Esch‐sur‐Alzette Luxembourg; ^7^ Disease Modeling and Screening Platform Luxembourg Institute of Systems Biomedicine, University of Luxembourg and Luxembourg Institute of Health Belvaux Luxembourg; ^8^ Department of Molecular Medicine University of Pavia Pavia Italy; ^9^ Metabolomics Platform, Enzymology and Metabolism Luxembourg Centre for Systems Biomedicine University of Luxembourg Esch‐sur‐Alzette Luxembourg; ^10^ Institute of Neurogenetics University of Lübeck Lübeck Germany; ^11^ Centre Hospitalier de Luxembourg Parkinson Research Clinic Luxembourg Luxembourg; ^12^ Transversal Translational Medicine Luxembourg Institute of Health Strassen Luxembourg

**Keywords:** Parkinson's disease, *PINK1*, isogenics, cyclodextrin, organoids

## Abstract

**Background:**

The etiology of Parkinson's disease (PD) is only partially understood despite the fact that environmental causes, risk factors, and specific gene mutations are contributors to the disease. Biallelic mutations in the phosphatase and tensin homolog (PTEN)‐induced putative kinase 1 (*PINK1*) gene involved in mitochondrial homeostasis, vesicle trafficking, and autophagy are sufficient to cause PD.

**Objectives:**

We sought to evaluate the difference between controls' and *PINK1* patients' derived neurons in their transition from neuroepithelial stem cells to neurons, allowing us to identify potential pathways to target with repurposed compounds.

**Methods:**

Using two‐dimensional and three‐dimensional models of patients' derived neurons we recapitulated PD‐related phenotypes. We introduced the usage of midbrain organoids for testing compounds. Using Clustered Regularly Interspaced Short Palindromic Repeats (CRISPR)/CRISPR‐associated protein 9 (Cas9), we corrected the point mutations of three patients' derived cells. We evaluated the effect of the selected compound in a mouse model.

**Results:**

PD patient‐derived cells presented differences in their energetic profile, imbalanced proliferation, apoptosis, mitophagy, and a reduced differentiation efficiency to tyrosine hydroxylase positive (TH+) neurons compared to controls' cells. Correction of a patient's point mutation ameliorated the metabolic properties and neuronal firing rates as well as reversing the differentiation phenotype, and reducing the increased astrocytic levels. Treatment with 2‐hydroxypropyl‐β‐cyclodextrin increased the autophagy and mitophagy capacity of neurons concomitant with an improved dopaminergic differentiation of patient‐specific neurons in midbrain organoids and ameliorated neurotoxicity in a mouse model.

**Conclusion:**

We show that treatment with a repurposed compound is sufficient for restoring the impaired dopaminergic differentiation of PD patient‐derived cells. © 2021 The Authors. *Movement Disorders* published by Wiley Periodicals LLC on behalf of International Parkinson and Movement Disorder Society

Neurodegenerative diseases pose a great threat to aging populations.^1^ Due to the lack of disease‐modifying therapies, patients suffering from Parkinson's disease (PD) have to rely on symptomatic treatments.^2^ Affected genes, reported as risk or causative factors of PD, control major cellular processes such as cell proliferation, membrane trafficking, mitochondrial homeostasis, and autophagy.^3^ Among these, *PINK1* is involved in regulating mitochondrial function and morphology by quarantining damaged mitochondria before their degradation as well as triggering the process of mitophagy.^4^ The fact that individuals with biallelic pathogenic variants in *PINK1* develop PD shows that an altered mitochondrial function, morphology, and degradation are linked to its pathogenesis.^5^

One of the hallmarks of PD is the loss of dopaminergic neurons in the substantia nigra pars compacta, but other regions are also affected.^6^ Reports of postmortem neuropathological studies of patients having mutations in *PINK1* show that Lewy bodies, mainly composed of synuclein alpha (SNCA), are present in several brain regions of some of these patients.^7^ Work in the zebrafish and organoid models previously suggested that a loss of function of *PINK1* might lead to a developmental reduction in the number of dopaminergic neurons.^8^, ^9^ However, implications of *PINK1* and its PD‐associated mutations during the transition from neural precursor cells to differentiated neurons were not studied in depth in a human cell model. Techniques for recapitulating disease phenotypes in culture had a recent breakthrough with the introduction of organoid cultures.^10^ These organ‐like structures contain different cell types in a spatially organized fashion, recapitulating at least some of the main functions of the respective organ. Importantly, they have been proven valid models for human diseases.^10^, ^11^, ^12^

In this study, the induced pluripotent stem cells (iPSCs) of patients with PD with *PINK1* mutations and healthy individuals were differentiated into a neuroepithelial stem cell (NESC) state and later into dopaminergic neurons. Different features such as proliferation capacity, apoptosis, and differentiation efficiency were analyzed using computational algorithms for pattern recognition through high‐content image analysis. We demonstrated that the differentiation efficiency of patient‐derived NESCs is reduced while maintaining an increased proliferative activity on neuronal differentiation and exhibiting increased apoptosis of tyrosine hydroxylase positive (TH+) neurons. We performed an immuno‐based protein‐profiling analysis of organoids at different stages of development confirming that proteins involved in cell cycle, differentiation, apoptosis, and autophagy pathways were differentially abundant in the case of the patient. Using extracellular flux analysis and microelectrode array, we assessed the energetic profile of NESCs and the firing activity of differentiated neurons, which were altered in patient‐derived cells. Moreover, using a pH‐sensitive reporter tagging a mitochondrial protein, we observed a reduced autophagy and mitophagy capacity. Gene correcting the patients' mutation with the clustered regularly interspaced short palindrome repeats (CRISPR)/CRISPR‐associated protein 9 (CRISPR/Cas9) system improved the mitochondrial activity, firing rate, and differentiation efficiency. Treatment with the compound 2‐hydroxypropyl‐β‐cyclodextrin (HP‐β‐CD) resolved the mitophagy impairment and improved the dopaminergic differentiation in patient‐derived cells by modifying the levels of proteins involved in dopaminergic differentiation, autophagy, apoptosis, and neuroinflammation.

## Materials and Methods

### Reagents and Resources Information

Detailed information about the reagents and resources used in this article are summarized in Table S7.

### Detailed Description of Protocols

A summary is given of the different procedures. A detailed explanation can be found in the Supplementary Information, Extended Material and Methods.

### Information About Cell Lines

Healthy controls 1 and 2 gave written informed consent at the University of Tübingen. Healthy control 3 was provided by Bill Skarnes. Patient *PINK1* 1 and patient *PINK1* 2 gave written informed consent at the University of Lübeck. Patient *PINK1* 3 and patient parkin samples were obtained from Coriell Institute, now held by the National Institute of Neurological Disorders and Stroke human cell and data repository. From each donor, one clone per iPSC line was derived and used in this study.

### NESC Derivation and Neuron Differentiation

Human NESCs were generated as described elsewhere.^13^ Neuronal differentiation was induced by culturing NESCs in N2B27 supplemented with 10 ng/mL human Brain Derived Neurotrophic Factor (hBDNF) (Peprotech, 450‐02), 10 ng/mL human Glial cell line‐derived Neurotrophic Factor (hGDNF) (Peprotech [East Windsor, NJ], 450‐10), 500 μM Dibutyryl Cyclic Adenosine Monophosphate (dbcAMP) (Sigma [St. Louis, MO], D0627), 200 μM ascorbic acid, 1 ng/mL Transforming growth factor beta 3 (TGF‐β3) (Peprotech, 100‐36E), and 1 μM purmorphamine (PMA) (differentiation media 1) for 6 days. Afterward, the same media without PMA (differentiation media 2) was used for the duration of the correspondent experiment.

### Immunocytochemistry

Fixation was done using 4% Paraformaldehyde (PFA) for 15 minutes at Room Temperature (RT). After 3× 1 × phosphate‐buffered saline (PBS) washing steps, cells were permeabilized using 0.5% Triton X‐100 in 1 × PBS for 15 minutes at RT. Blocking was performed for 1 hour at RT. Incubation with the first antibodies was done overnight at 4°C in blocking buffer. Incubation with the secondary antibodies was for 2 hours at RT in blocking buffer.

### Immunohistochemistry

Processing of organoids was performed as previously described.^14^

### Western Blotting

Pellets of neurons differentiated for 21 days coming from a confluent well of a 6‐well plate were lysed. Lysates were then centrifuged, quantified, resolved, and transferred from the gel to polyvinylidene fluoride membranes in an iBlot2 device (Thermo Fisher [Waltham, MA], IB24001). Membranes were blocked for 60 minutes at RT. Primary antibodies were incubated at 4°C overnight. Secondary antibodies were incubated for 60 minutes at RT. Membranes were revealed using the SuperSignal West Pico Chemiluminescent Substrate (Thermo Fisher [Waltham, MA]).

### Extracellular Flux Analysis (SeaHorse Measurements)

Human NESCs were seeded in a Matrigel‐coated XF 96‐well plate (Agilent Technologies [Santa Clara, CA], 102416‐100) at a density of 65,000 cells per well. Three baseline measures and three measurements after each compound injection were performed.

### Microelectrode Array Measurements

The Maestro microelectrode array (MEA; Axion BioSystems [Atlanta, GA]) system was used to measure the spontaneous activity of neurons. Axion Integrated Studio was used to process the raw data as previously described.^14^

### Rosella Mitophagy Reporter

The pH sensor fluorescent protein pH‐sensitive green fluorescent protein (pHluorin) was fused to red fluorescent protein from Discosoma (DsRed) and the entire open reading frame of adenosine triphosphate (ATP) Synthase F1 Subunit Gamma (ATP5C1) or Microtubule‐associated protein 1A/1B‐light chain 3 (LC3) as described in Sargsyan et al^15^ and Arias‐Fuenzalida et al.^16^

### Gene Editing

Gene correction of patient's point mutation was performed as previously described.^17^, ^18^ Briefly, donor constructs with a positive selection module (PSM) and designed guide RNAs (gRNAs) targeting *PINK1* were transfected into human induced pluripotent stem cells (hiPSCs). Fluorescent selection was done by cell sorting; removal of the PSM was performed with transposase piggyBac excision‐only mRNA, and selection was done via cell sorting.

### RNA Isolation, Reverse‐Transcription Polymerase Chain Reaction, and Quantitative Polymerase Chain Reaction

Total RNA was isolated using miRNeasy Mini Kit (Qiagen, Hilden, Germany) and treated with the RNase‐Free DNase Set (Qiagen). cDNA was reverse transcribed using the High‐Capacity RNA‐to‐cDNA Kit (Invitrogen, Waltham, MA). Quantification of gene expression was performed using the LightCycler 480 Probes software (Roche, Basel, Switzerland).

### Compound Treatment

HP‐β‐CD dissolved in water (Sigma, H‐107) was added on every media change at the different concentrations tested and kept throughout the entire differentiation process. Untreated conditions are regular differentiation media.

### Image Acquisition

Cell carrier Ultra plates were imaged in an automated manner using an Opera Quadruple Enhanced High Sensitivity (QEHS) spinning disk microscope (PerkinElmer, Waltham, MA).

### Image Analysis

The image analysis was performed using MatLab (MathWorks, Natick, MA) as previously described.^16^, ^19^

### Microfluidics Culture

Neuroepithelial stem cells (NESCs) were seeded in an OrganoPlate (Mimetas [Leiden, the Netherlands], 9603‐400‐B) as explained elsewhere.^20^

### Immuno‐Based Protein‐Profiling Sample Incubation, Data Acquisition, and Analysis

Organoids from control and patient‐derived NESCs were treated with HP‐β‐CD in a 96‐well ultra‐low attachment (ULA) plate format for 30 days of differentiation at a concentration of 5 μM. The samples were analyzed on scioDiscover antibody microarrays (Sciomics, Baden‐Wuerttemberg, Germany) targeting 1360 different proteins with 1830 antibodies. Differences in protein abundance or phosphorylation levels between different samples or sample groups are presented as log‐fold changes (logFC) calculated for the basis two.

### Animal Experiments

A total of 15 male, 2‐month‐old C57bl/6 mice were used in the study. MPTP at 30 mg/kg was intraperitoneally injected for 5 days to generate the subacute PD mouse model. Model verification was performed using both behavioral analysis and pathological assessment. TH staining was used to evaluate the dopaminergic neuronal loss in substantia nigra. HP‐β‐CD was injected every second day in the dose of 4000 mg/kg subcutaneously for 15 days.

### Statistical Analysis and Graphical Representation

Statistical analysis performed on each assay is mentioned in each figure legend. All of the statistical analyses were performed in R (R Foundation for Statistical Computing, Vienna, Austria). Clustering and heatmaps were produced using the complexheatmap package of R.^21^

### Results

#### Reduced TH+ Neuron Differentiation, Increased Proliferation, Astrocyte Activation, and Apoptosis in Patient‐Specific Cells

**FIG 1:**
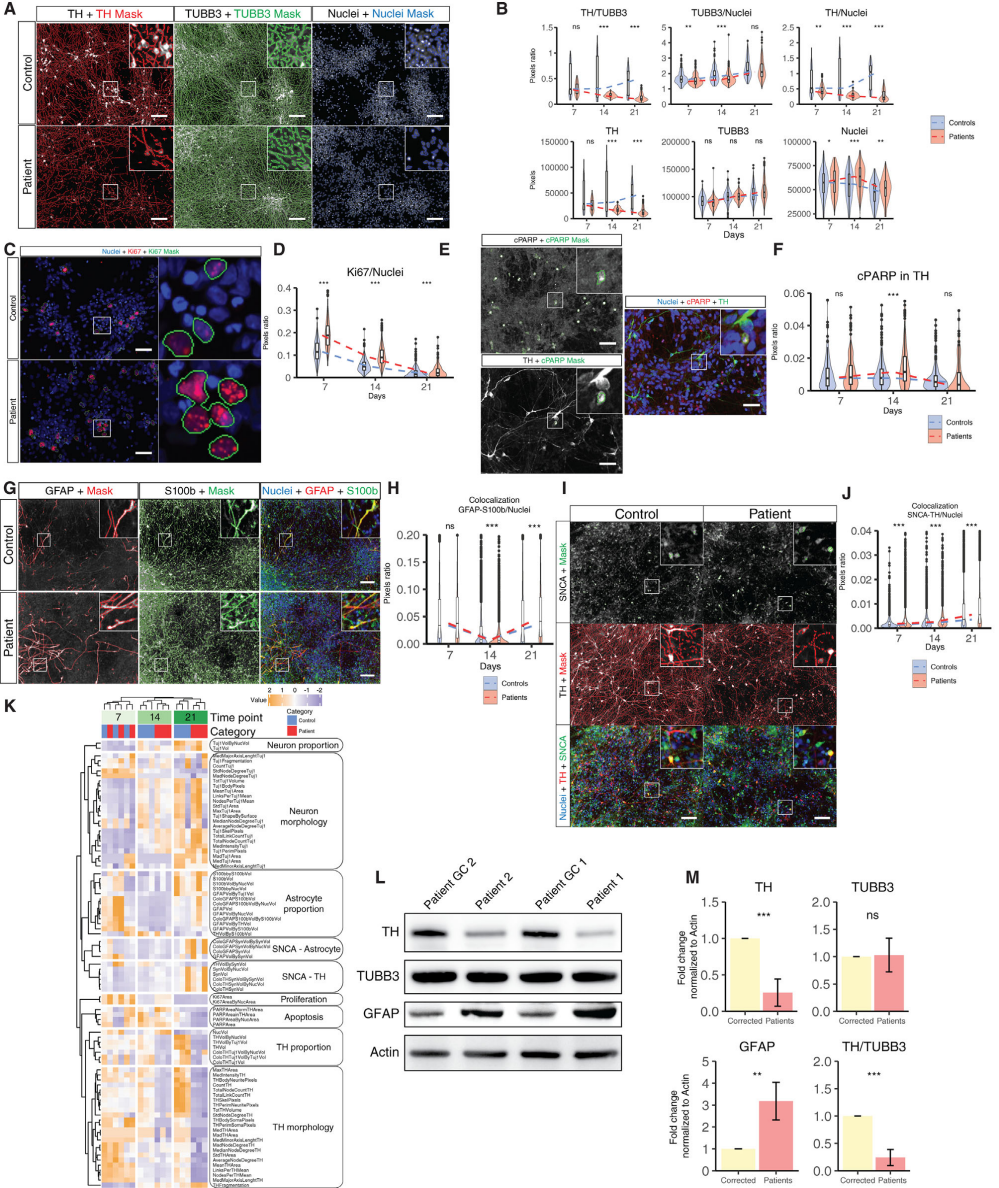
Impaired differentiation of neural stem cells of patient carrying *PINK1* mutations. (**A**) Images representing the median values of a 14‐day differentiation neuronal two‐dimensional culture of controls and patients groups. Raw images of the markers tyrosine hydroxylase (TH), Tubulin Beta 3 Class III (TUBB3), and Hoechst are presented with its respective perimeter mask and a zoomed region (scale bar = 100 μm). (**B**) Quantification of TH, TUBB3, and Hoechst at time points 7, 14, and 21 after the induction of differentiation. Pixel quantification (lower panel) with their respective ratios (upper panel). Acquisition was performed at 20× sampling randomly 15 fields per well. Five wells of controls and five wells of patients were acquired per replicate. Total fields of controls (fc) and fields of patients (fp) analyzed time points 7 (fc = 219; fp = 215), 14 (fc = 209; fp = 219), and 21 (fc = 207; fp = 220) were collected over three independent replicates using all the lines. (**C**) Images representing the median values of proliferation marker Ki67 and Hoechst of control and patient‐derived cells at day 7 of differentiation in a two‐dimensional culture with their respective zoomed region and perimeter mask (scale bar = 50 μm). (**D**) Quantification of Ki67 at time points 7, 14, and 21 after the induction of differentiation in a two‐dimensional culture, normalized to the nuclear area. Acquisition was performed at 20× sampling randomly 15 fields per well. Ten wells of controls and 10 wells of patients were acquired per replicate per time point. Images analyzed per time point: 7 (fc = 425; fp = 421), 14 (fc = 411; fp = 424), and 21 (fc = 416; fp = 432) were collected over three independent replicates using all the lines. (**E**) Representative images of apoptotic marker cleaved poly adenosin phosphate (ADP)‐ribose polymerase (cPARP), dopaminergic marker TH, and Hoechst at day 14 of differentiation in a two‐dimensional culture with their respective zoomed region and perimeter mask (scale bar = 50 μm). (**F**) Quantification of cPARP within the TH area at time points 7, 14, and 21 after the induction of differentiation in a two‐dimensional culture, normalized to the TH area. Acquisition was performed at 20× sampling randomly 15 fields per well. Ten wells of controls and 10 wells of patients were acquired per replicate per time point. Images analyzed per time point: 7 (fc = 219; fp = 215), 14 (fc = 209; fp = 219), and 21 (fc = 207; fp = 220) were collected over three independent replicates using all of the lines. (**G**) Images representing the median values of a 21‐day differentiation neuronal two‐dimensional culture of control and patient groups. Raw images of the markers glial fibrillary acidic protein (GFAP) and S100 calcium binding protein B (S100b) are presented with their respective perimeter masks and zoomed regions (scale bar = 100 μm). (**H**) Quantification of colocalization between GFAP, S100B, and TH normalized to nuclear area at time points 7, 14, and 21 after the induction of differentiation in a two‐dimensional culture. (**I**) Images representing the median values of a 21‐day differentiation neuronal two‐dimensional culture of control and patient groups. Raw images of the markers synuclein alpha (SNCA) and TH are presented with their respective perimeter masks and zoomed regions (scale bar = 100 μm). (**J**) Quantification of colocalization between SNCA and TH normalized to nuclear area at time points 7, 14, and 21 after the induction of differentiation in a two‐dimensional culture. (**K**) Heatmap clustering different phenotypes during the process of differentiation between control and patient‐derived neurons in a two‐dimensional culture. Normalized scale within category of phenotype. (**L**) Western blot analysis of TH, TUBB3, and GFAP proteins extracted from two patient‐derived *PINK1* p.Q456X‐mutant neurons (patients 1 and 2) and their respective isogenic gene‐corrected (GC) controls (patient 1 GC and patient 2 GC) after 30 days of differentiation (two‐dimensional cultures). β‐actin was used as loading control. (**M**) Quantitative immunoblot analysis of data presented in **C**. Histogram bars represent the mean values (± standard deviation) of TH, TUBB3, and GFAP signals in at least three independent experiments using the two isogenic pairs. Data were normalized against β‐actin levels and expressed as fold change. Except for panels **L** and **M**, all control and patient lines were used. Statistical analysis was performed using Kruskal–Wallis and Dunn's tests for multiple comparisons. Adjustment of the *P*‐value for multiple tests was performed using Benjamini‐Hochberg. *P < 0.05, **P < 0.01, ***P < 0.001; ns, not significant. [Color figure can be viewed at wileyonlinelibrary.com]

To evaluate whether impaired mitochondrial function could affect dopaminergic differentiation, hiPSCs were derived from three patients carrying a mutation in *PINK1*: two carrying p.Q456X (rs45539432)^22^ and one carrying p.I368N (rs774647122) and three age‐matched and sex‐matched controls (Fig. S1A). Human iPSCs were further differentiated into a stable neural precursor state (NESCs) following a previous report^13^ and used as a starting population for studying dopaminergic neuron differentiation efficiency (Fig. S1B). Using an automated image analysis algorithm, the proportion of Tubulin Beta 3 Class III (TUBB3)–positive signal that colocalizes with TH was quantified. Patient‐derived NESCs showed a reduced capacity to differentiate into TH+ neurons (worsened at later time points) while maintaining the same level of overall neuronal differentiation compared with controls (Fig. 1A,B, Table S8). To confirm the impaired dopaminergic differentiation, we differentiated NESCs in a three‐dimensional microfluidic environment observing the same pattern (Fig. S1C,D). Assessment of the proliferation marker Ki67 after 7, 14, and 21 days of differentiation (Fig. 1C–F) showed that patient‐derived cells maintained a higher proliferative capacity after induction of differentiation. The apoptotic marker cleaved poly adenosin phosphate (ADP)‐ribose polymerase (cPARP) showed an increased signal in patient‐derived TH+ neurons at time point 14 (Fig. 1E,F). The amount of glial fibrillary acidic protein (GFAP) and S100 calcium binding protein B (S100b)–positive cells (markers of activated astrocytes) was significantly higher at day 21 of differentiation (Fig. 1G,H), the time point with the lowest TH levels in patients. During differentiation, there is a reduction of the GFAP marker at day 14 (controls being higher) followed by an increase at day 21 (higher increase in patient cells). An increase in astrocytic markers have been previously linked to oxidative stress.^23^ Total levels of SNCA colocalizing with TH significantly increased in the patients through the different stages of differentiation (Fig. 1I,G). A cluster analysis considering all the different phenotypic features analyzed through the different time points of differentiation showed a discerning pattern in TH proportion and morphology at later time points, days 14 and 21 of differentiation (Fig. 1K).

#### Gene Correction–Restored Energetic Profile and Differentiation Efficiency

To evaluate the effect of the point mutation in cells derived from patients with PD, homozygous correction of the g.20655C>T (p.Gln456Ter) mutation in *PINK1* was performed in two patient lines and homozygous correction of the c.1103T>A (p.Ile368Asn) in one patient line using Fluorescence‐activated cell sorting (FACS)‐assisted CRIPSR/Cas9 editing.^17^, ^18^ After gene correction, the reduced TH levels were improved, and the increased levels of astrocytes were reduced in neurons cultured in two‐dimensional conditions (Fig. 1L,M). The increased levels of TH after gene correction were also confirmed by immunofluorescence (Fig. S2A,B). Extracellular flux analysis (Seahorse, Agilent Technologies [Santa Clara, CA]) showed that gene correction reduced significantly the higher glycolytic activity of patient‐derived NESCs (Fig. S2C–E,F). MEA measurements showed that the firing activity and the network burst firing in neurons cultured in two‐dimensional were increased after gene correction (Fig. S2F–H). To assess the gene correction of *PINK1* not only at the genomic level but also at a functional level, we analyzed the expression of *PINK1*‐regulated mitophagy markers in differentiated neurons cultured in two‐dimensional. Mono‐ubiquitination of Voltage Dependent Anion Channel 1 (VDAC1), performed by parkin in a *PINK1*‐dependent manner,^24^ was restored in the two gene‐corrected lines tested. This was accompanied by decreased parkin levels in Carbonyl cyanide m‐chlorophenyl hydrazone (CCCP)‐treated neurons after *PINK1* gene correction as a result of mitophagy‐induced parkin degradation^25^ (Fig. S2I,J).

#### Proteomics Analysis in Organoids Confirm Dysregulated Pathways at Different Time Points

**FIG 2:**
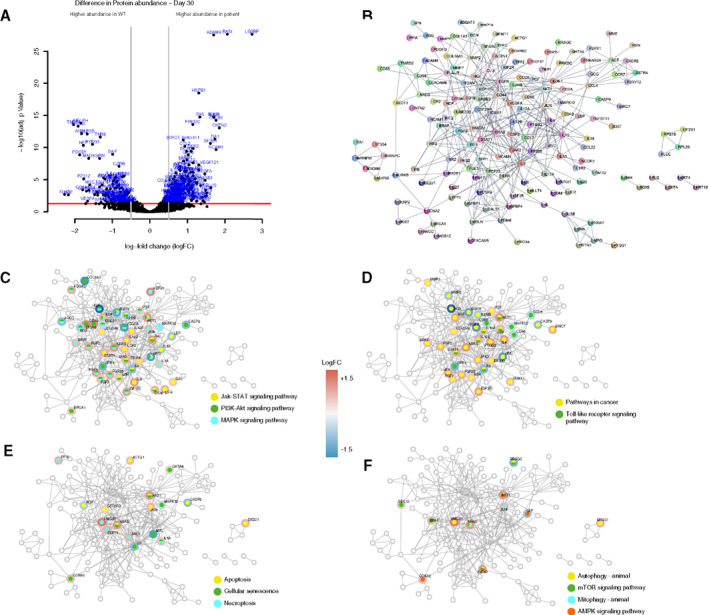
Differential abundance of proteins between control and patient‐derived organoids at day 30 of neuronal differentiation. (**A**) Volcano plot of proteomics data. The *x* axis represents the log fold change (logFC) between patient‐derived and control organoids, with positive logFC indicating that the protein is more abundant in patient data than in control data, and the opposite for negative logFC. The *y* axis represents the *P*‐value of the comparison adjusted for multiple testing using Benjamini‐Hochberg. Proteins with adjusted *P*‐value <0.05 and absolute logFC >0.5 were considered differential. (**B**) Network of the protein–protein interactions among the differential proteins obtained from the Search Tool for the Retrieval of Interacting Genes/Proteins (STRING) database. Interactions obtained from all data sources and with a confidence score >0.9 (high) were considered. Differential proteins that are not reported to interact with other differential proteins are not represented. (**C**–**F**) Mapping of significantly enriched Kyoto Encyclopedia of Genes and Genomes (KEGG) pathways on the protein–protein interaction network. KEGG pathways were tested for enrichment in proteins present in the network compared to the human genome and were considered significantly enriched if their *P*‐value adjusted by Benjamini‐Hochberg was <0.05. Nodes corresponding to proteins that belong to a selection of significantly enriched pathways are highlighted with different colors on the STRING network. The border of the nodes depicts the logFC of the pathway proteins in the comparison of control and patient‐derived organoids. Control line 1 and patient line 1 were used for the proteomics experiments. Jak‐STAT, janus kinase and signal transducer and activator of transcription; PI3K‐Akt, phosphatidylinositol 3‐kinase and Akt (protein kinase B); MAPK, mitogen‐activated protein kinase; mTOR, mechanistic target of rapamycin; AMPK, AMP‐activated protein kinase [Color figure can be viewed at wileyonlinelibrary.com]

We performed an immuno‐based protein analysis of three‐dimensional midbrain organoids derived from control and patient cells at different time points of differentiation. Differentially abundant proteins were detected at days 10 (182 proteins), 20 (302), and 30 (267) of differentiation (Fig. 2A, Fig. [Link], [Link], and Supplemental Datasets 1–3). Selected protein–protein interaction analysis using the Search Tool for the Retrieval of Interacting Genes/Proteins (STRING) database^26^ and Cytoscape^27^ revealed several direct as well as indirect interactions of the differential proteins corresponding with pathways involved in apoptosis, necroptosis, protein synthesis, metabolism, and cell cycle (Fig. 2B–F). Also, proteins involved in inflammation and autophagy showed dysregulation between controls and patients during the process of differentiation (Fig. 2E,F and Fig. S3).

#### *PINK1* Patient‐Specific Neurons Present a Reduced Mitophagy Capacity

**FIG 3:**
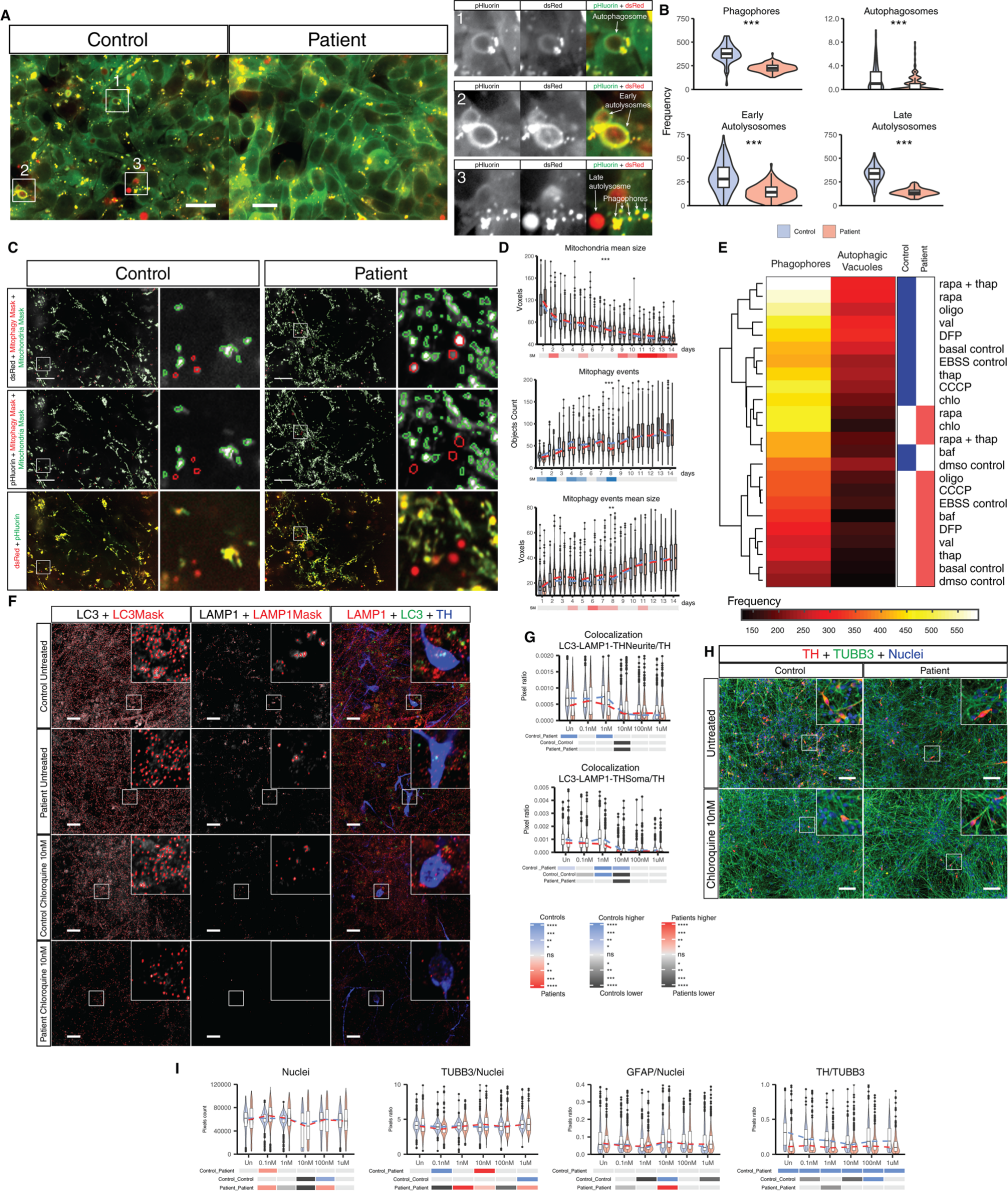
Modulation of autophagy alters neuronal differentiation. (**A**) Representative images of human induced pluripotent stem cells (hiPSCs) carrying the Rosella construct targeting microtubule‐associated proteins 1A/1B light chain 3B (LC3) for control and patient‐derived cells and zoomed images with representative identification of the different stages of the autophagy process detected with the Rosella reporter (scale bar = 20 μm). (**B**) Absolute quantification of phagophores, autophagosomes, early autolysosomes, and late autolysosomes for controls and patient‐derived hiPSCs. All structures were measured under basal conditions. Acquisition was performed at 60× sampling randomly. Images analyzed: fields of controls (fc) = 131 and fields of patients (fp) = 131 were collected over three independent replicates using control line 1 and patient line 3. (**C**) Images representing the median values of neurons in a two‐dimensional culture tagged with the Rosella construct for depicting mitophagy events at day 8 of differentiation showing the red fluorescent protein from Discosoma (dsRed) and pH‐sensitive green fluorescent protein (pHluorin) raw signal (with their corresponding masks). A merged image of both channels is shown at the bottom of the panel and zoomed images in the right panel of each line (scale bar = 20 μm). (**D**) Time series quantification of the mitophagy capacity during neuronal differentiation for 14 days in a two‐dimensional culture. Different properties of mitochondria and mitophagy events were assessed. Measurements were performed once a day during the entire differentiation protocol. Images analyzed: fc = 97–219 and fp =126–224 range measured per day for 14 days. Acquisition was performed at 60× sampling randomly 15 fields per well. Five wells of control 1 and 5 wells of patient 3 were acquired per replicate over three independent replicates. (**E**) Heatmap clustering for control and patient‐derived cells across all mitophagy and autophagy modulating treatments in control line 1 and patient line 3 hiPSCs. Scale in absolute event frequency of phagophores or autophagic vacuoles detected. (**F**) Images representing the median values of neurons in a two‐dimensional culture stained for LC3, lysosomal associated membrane protein 1 (LAMP1), and tyrosine hydroxylase (TH) after treatment with different concentrations of chloroquine with their respective zoomed areas (scale bar = 20 μm). (**G**) Quantification of immunostaining for LC3, LAMP1, and TH+ and their respective colocalizations, normalized to nuclear area at different chloroquine concentrations. (**H**) Images representing the median values of neurons in a two‐dimensional culture stained for Tubulin Beta 3 Class III (TUBB3), glial fibrillary acidic protein (GFAP), and TH after treatment with different concentrations of chloroquine, with their respective zoomed areas (scale bar = 50 μm). (**I**) Quantification of immunostaining for GFAP, TUBB3, and TH+ and their respective colocalizations, normalized to nuclear area at different chloroquine concentrations. Except for panels **A** to **E**, all control and patient lines were used. Statistical analyses for panels **B**, **G,** and **I** were performed using Kruskal–Wallis and Dunn's tests for multiple comparisons. Statistical analysis for panel **D** was performed using a nonparametric test for repeated measures in factorial design (nparLD). Adjustment of the *P*‐value for multiple tests was performed using Benjamini‐Hochberg. *P < 0.05, **P < 0.01, ***P < 0.001, ****P < 0.0001; ns, not significant. baf, bafilomycin; CCCP, carbonyl cyanide m‐chlorophenyl hydrazone; chlo, chloroquine; DFP, deferiprone; dmso, Dimethyl sulfoxide; EBSS, Earle's Balanced Salt Solution; oligo, oligomycin; rapa, rapamycin; thap, thapsigargin; val, valinomycin. [Color figure can be viewed at wileyonlinelibrary.com]

To understand the mitochondrial dynamics in these cells, we generated lines expressing the Rosella construct bound to microtubule‐associated proteins 1A/1B light chain 3B (LC3) or to adenosine triphosphate (ATP) synthase F1 subunit gamma (ATP5C1) to evaluate the autophagy status and mitochondrial degradation by mitophagy (Fig. 3). Already at the hiPSC level, classification of the different stages of autophagy^16^ showed that autophagy was reduced in patient‐derived cells (*PINK1*, p.I368N) compared with a control line (Fig. 3A,B). To confirm with a different assay, we analyzed the ratio of LC3 membrane‐bound form/LC3 cytosolic form (LC3‐II/LC3‐I) by Western blot, which was reduced in 21‐day differentiated neurons in two‐dimensional cultures derived from patients (Fig. S6A,B). Measurements throughout differentiation revealed significantly fewer mitophagy events in patients' neurons carrying the Rosella construct (Fig. 3C,D and Fig. S6C). The mitophagy events observed in *PINK1* patient‐derived cells might be mediated by *PINK1*‐parkin independent mitophagy pathways.^28^ Rapamycin inhibits the mammalian target of rapamycin (mTOR), which modulates autophagy by reducing transcription factor EB (TFEB)'s nuclear translocation.^29^ Patient‐derived cells treated with rapamycin showed an increase in the frequency of phagophores and autophagic vacuoles to similar levels as those observed in controls, which presented a similar response to the rapamycin treatment (Fig. 3E and Fig. S6D). In this study, one patient and control line were genetically modified to stably carry the Rosella construct, hence caution is needed in interpreting the mitophagy results detected with the reporter. We then assessed the effect of chloroquine, a known blocker of the autophagic flux by reducing the fusion between autophagosomes and autolysosomes,^30^ during the neuronal differentiation process. The total levels of LC3 were increased in the patient‐untreated group and reduced after treatment with chloroquine (Fig. S6E). No difference was observed in the total levels of lysosomal associated membrane protein 1 (LAMP1) in untreated samples. When evaluating the levels of colocalization of LC3 and LAMP1, no difference in the overall colocalization was observed, but when considering the colocalization with TH as well, control TH+ neurons presented a higher level of colocalized LC3‐LAMP1 in the soma as well as in the neurites in untreated conditions (Fig. 3F,G). This occurred concomitantly with a significant reduction in the levels of TH+ cells at a concentration of 10 nM of chloroquine and increased GFAP particularly in controls (Fig. 3H,I and Fig. S6F). Higher concentrations of chloroquine showed an increase of the TH+ levels compared with the treatment with 10 nM chloroquine, which could be explained by the reported increase of transforming growth factor beta (TGFβ) superfamily receptors in the plasma membrane after chloroquine treatment.^31^ TGFβ plays an important role in the specification of dopaminergic neurons.^32^, ^33^, ^34^

#### Treatment with HP‐β‐CD Increased TFEB Nuclear Translocation in Neurons, Modified the Abundance of Proteins Related to Dopaminergic Neurons Differentiation, and Increased Dopaminergic Neuron Differentiation Efficiency of Brain Organoids

**FIG 4:**
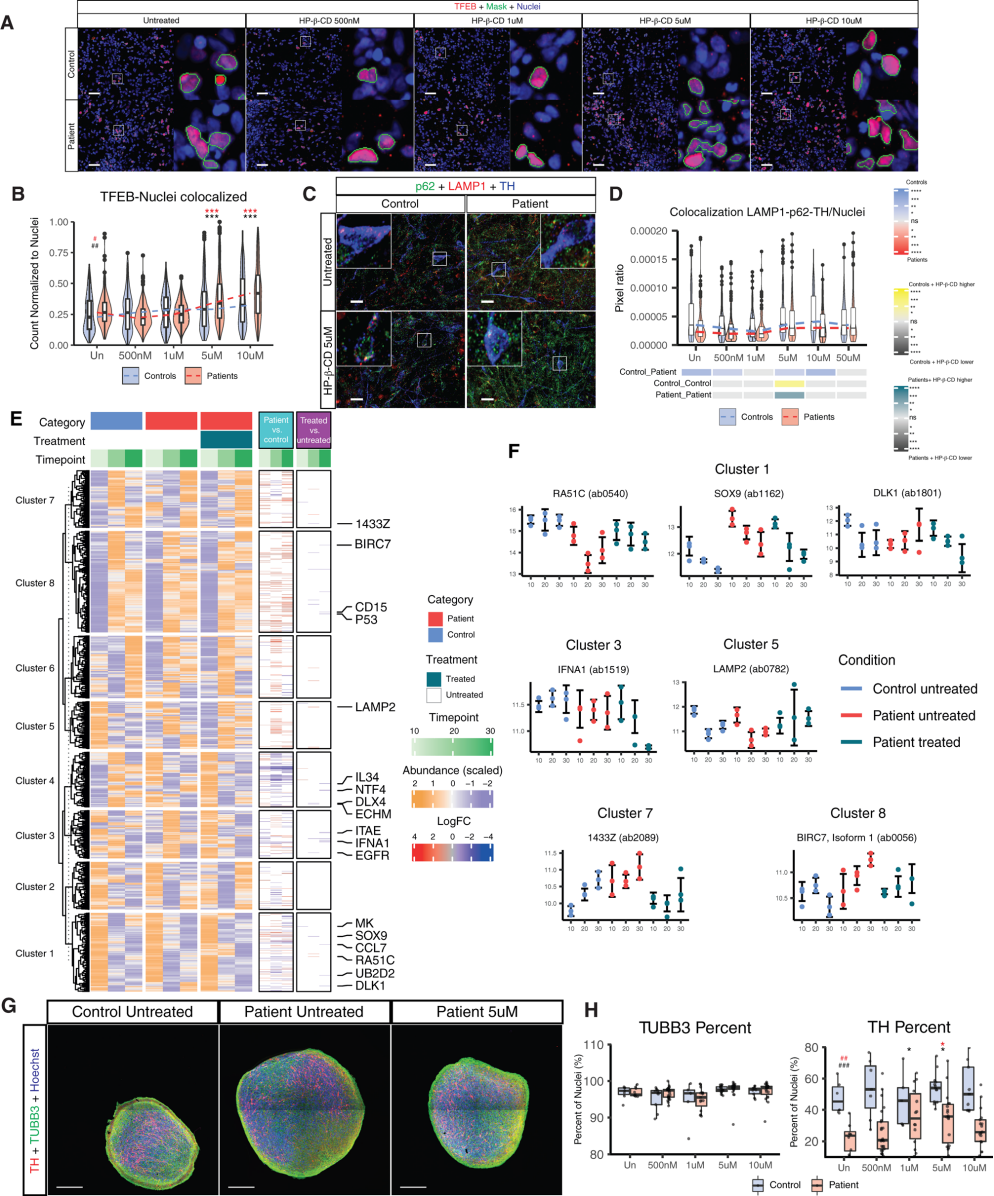
Treatment with HP‐β‐CD improves neuronal differentiation by increasing autophagy. (**A**) Representative images of differentiated neurons in a two‐dimensional culture stained for TFEB, with their respective zoomed images (scale bar = 50 μm). (**B**) Quantification of the colocalization between TFEB and Nuclei signal with the different treatment concentrations. Images analyzed: 540 fields per category (control or patient) per condition, acquired over three independent replicates using all lines. (**C**) Images representing the median values of neurons in a two‐dimensional culture stained for p62, lysosomal associated membrane protein 1 (LAMP1), and tyrosine hydroxylase (TH) after treatment with different concentrations of HP‐β‐CD, with their respective zoomed areas (scale bar = 20 μm). (**D**) Quantification of immunostaining for the colocalization of sequestosome 1 (SQSTM1 or p62), LAMP1 and TH+, normalized to nuclear area at different HP‐β‐CD treatment concentrations. (**E**) Protein abundance measured in control‐derived, patient‐derived, and HP‐β‐CD‐treated patient‐derived organoids at time 10, 20 and 30 days of the neuronal differentiation was scaled for each protein separately. A k‐means partitioning (k = 8) was performed in order to obtain clusters of proteins with similar expression dynamics. The proteins differentially abundant between control and patient‐derived organoids, and between patient and treated patient‐derived organoids are also shown (Benjamini‐Hochberg, BH‐adjusted *P*‐value<0.05 and absolute logFC>0.5). (**F**) Normalized expression of selected proteins that show differential expression between patient and treated patient‐derived organoids is reported. Three data points were collected for each condition and time point. Whiskers represent one standard deviation from the median of the measurements. (**G**) Representative images of control, patient and patient treated derived organoids at 30 days of differentiation (scale bar = 200 μm). (**H**) Quantification of the markers TH, Tubulin Beta 3 Class III (TUBB3) and Hoechst. Each dot represents one section analyzed. Sections analyzed: control (7, 9, 5, 14, and 9) and patient (9, 25, 15, 18, and 19) respectively for the different treatments (Un, 500 nM, 1 μM, 5 μM, and 10 μM) collected over three independent replicates. Control 1, patient 1, and patient 3 lines were used. For panels **A** to **D**, all control and patient lines were used. For panels **E** and **F**, control line 1 and patient line 1 were used. For panels **B**, **D,** and **H**, statistical analyses were performed using Kruskal–Wallis and Dunn's tests for multiple comparisons. Adjustment of the *P*‐value for multiple tests was performed using Benjamini‐Hochberg (BH). The adjusted significance are represented in red. Comparisons between control untreated and patient untreated are presented with #. Comparison between the patient‐untreated condition and the different treatment concentrations are represented with *. *P < 0.05, **P < 0.01, ***P < 0.001, ****P < 0.0001; ns, not significant. Significance hashtag represent: #*P* < 0.05, ##*P* < 0.01, ###*P* < 0.001; ns stands for not significant. Un, untreated. 1433Z, tyrosine 3‐monooxygenase/tryptophan 5‐monooxygenase; BIRC7, baculoviral inhibitor of apoptosis protein (IAP) repeat containing 7; CCL7, C‐C motif chemokine ligand 7; CD15, fucosyltransferase 4; DLK1, Delta like non‐canonical notch ligand 1; DLX4, distal‐less homeobox 4; ECHM, enoyl coenzyme a hydratase short chain 1 mitochondrial; EGFR, epidermal growth factor receptor; IFNA1, interferon alpha 1; IL34, interleukin 34; ITAE, integrin subunit alpha E; LAMP2, lysosomal associated membrane protein 2; MK, midkine; NTF4, neurotrophin 4; P53, tumor protein 53; RADC, RAD51 paralog C; SOX9, sex‐determining region Y (SRY)‐box transcription factor 9; UB2D2, ubiquitin conjugating enzyme E2 D2 [Color figure can be viewed at wileyonlinelibrary.com]

Due to the observed altered autophagy and mitophagy pattern, we explored ways for modulating autophagy with repurposed compounds. Treatment with the compound HP‐β‐CD was reported to modulate autophagy by increasing TFEB nuclear translocation in human neuroglioma cells.^29^, ^35^, ^36^ Treatment with HP‐β‐CD during the entire differentiation process was able to increase the proportion of nuclei that colocalized with TFEB in a two‐dimensional neuronal culture at 21 days of differentiation (Fig. 4A,B). Moreover, HP‐β‐CD upregulated the gene expression of FK506 binding protein 8 (*FKBP8*) and FUN14 domain‐containing protein 1 (*FUNDC1*), outer mitochondrial membrane–anchored proteins that present the LC3 interacting region motif mediating mitophagy (Fig. S7A). Treatment with HP‐β‐CD led to an increase in mitophagy and in overall autophagy assessed with the Rosella construct (Fig. S7B,C). We also observed that this increased autophagy occurs in TH+ neurons by assessing the colocalization of sequestosome 1 (SQSTM1 or p62), LAMP1, and TH at concentrations above 5 μM reaching a plateau (Fig. 4C,D).

The effect of HP‐β‐CD during neuronal differentiation was assessed at three different time points in a three‐dimensional environment using midbrain organoids. Protein abundance analysis (Fig. 4E,F, Fig. S7D, Supplemental Datasets 1–6) showed differentially abundant proteins between untreated and treated patient‐derived organoids involved in the differentiation of dopaminergic neurons (SRY‐box transcription factor 9 [SOX9] and delta like non‐canonical notch ligand 1 [DLK1]), mitochondrial metabolism and mtDNA integrity (interferon alpha 1 [IFNA1] and RAD51 paralog C [RA51C]), and autophagy (lysosomal associated membrane protein 2 [LAMP2], tyrosine 3‐monooxygenase/tryptophan 5‐monooxygenase activation protein zeta [14‐3‐3ζ], and baculoviral IAP repeat containing 7 [BIRC7]) (Fig. 4F). The reduced proportion of dopaminergic neurons in patient‐derived brain organoids was increased after treatment with HP‐β‐CD increased the proportion of TH+ cells without changing the amount of TUBB3+ neurons (Fig. 4G,H, Fig. S8A, Table S8). HP‐β‐CD treatment in patient‐specific neurons, carrying the homozygous p.R275W (rs34424986) parkin RBR E3 ubiquitin protein ligase (*PRKN*) mutation, increased the amount of dopaminergic neurons (Fig. S8B) concomitant with an increase in nuclei pixels. Parkin is known to be a downstream effector of *PINK1* induction of mitophagy, and alterations can lead to mitochondrial alterations and TH+ neuronal loss.^37^ Because HP‐β‐CD composition is a mixture of cyclodextrin rings substituted with different degrees of hydroxypropyl,^38^ we further characterized the composition of the used HP‐β‐CD mixture in these experiments and determined that the most frequent isomer is one with six degrees of substitution (Fig. S9A–C). Knowledge of the cyclodextrin composition will leverage application in future clinical trials and facilitate comparisons of treatment results between different neurodegenerative diseases.^38^

#### Treatment with HP‐β‐CD Reduces the Toxicity Generated by ‐Methyl‐4‐Phenyl‐1,2,3,6‐Tetrahydropyridine (MPTP) Treatment in Mice

**FIG 5:**
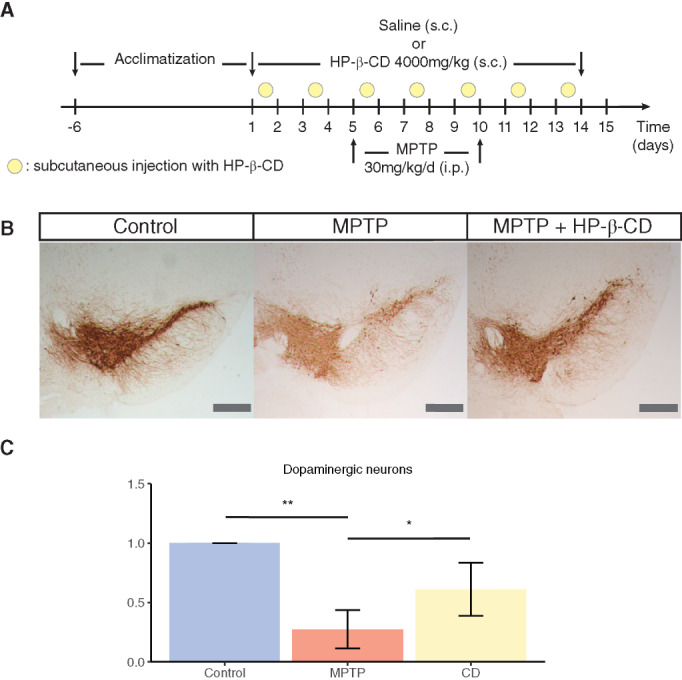
Treatment with 2‐hydroxypropyl‐β‐cyclodextrin (HP‐β‐CD) protects against toxicity of MPTP. (**A**) Treatment scheme for the generation of MPTP‐induced subacute Parkinson's disease mice model and treatment with HP‐β‐CD. (**B**) Representative mouse midbrain sections stained for tyrosine hydroxylase (TH) in control, MPTP, or HP‐β‐CD‐treated mice (scale bar = 400 μm). (**C**) Stereological quantification of the TH levels in mouse sections normalized to control levels. Statistical analysis was performed using Kruskal–Wallis and Dunn's tests for selected comparisons. *P < 0.05, **P < 0.01. [Color figure can be viewed at wileyonlinelibrary.com]

To assess the effect of HP‐β‐CD in in vivo conditions, 1‐methyl‐4‐phenyl‐1,2,3,6‐tetrahydropyridine (MPTP) induced subacute PD mice models were generated by intraperitoneal injection for 5 days of 30 mg/kg MPTP. A preventive treatment with HP‐β‐CD (Fig. 5A) was applied subcutaneously for 15 days. This treatment scheme showed a positive effect of HP‐β‐CD in reducing the loss of TH+ neurons in the midbrain of mice caused by MPTP (Fig. 5B,C).

## Discussion

Our findings suggest that a loss‐of‐function mutation in *PINK1* ^39^ affects the transition between a neural precursor state and a differentiated TH+ neuron (Fig. 1). Previous reports showed no difference between a patient line carrying the p.Q456X mutation and control lines; however, these differences could be explained by the different differentiation protocol^22^ and/or the low number of patient lines used in these studies.^40^ Patient‐derived cells remained highly proliferative upon induction of differentiation (Fig. 1C,D). This matches previous reports showing that the loss of *PINK1* activity triggers an increase in glycolysis via a reactive oxygen species–mediated stabilization of hypoxia‐inducible factor‐1α (HIF1α), associated with the Warburg effect.^41^, ^42^ Cells that manage to differentiate into TH+ neurons presented higher levels of apoptosis as previously reported.^43^, ^44^, ^45^ Patients' derived neurons also presented an increased proportion of activated astrocytes (Fig. 1G,H) as well as of total SNCA in TH+ cells (Fig. 1I,J). Gene correction of *PINK1* allowed neural precursor cells to reduce their dependence on glycolysis and increase the firing activity in differentiated neurons as well as increase the proportion of TH+ neurons upon differentiation (Fig. 1L,M, Fig. S2). Gene correction seems to trigger a metabolic change that allows the switch from stem cells to differentiated cells.^46^

Proteomics analysis of control and patient‐derived organoids at different stages of differentiation confirmed that the altered cell cycle, increased apoptosis, and reduced differentiation capacity observed coincided with dysregulation of these pathways (Fig. 2C–F, and Fig. S3A–D). It also showed dysregulation of the autophagy pathway, a common process impaired in PD as well as in other neurodegenerative diseases.^47^, ^48^

Impaired mitochondrial turnover, due to reduced mitophagy activity, was observed in patient cells (Fig. 3C,D) in accordance with a previous report.^49^ This altered mitophagy balance occurred simultaneously with an overall impaired autophagy (Fig. 3A,B,G). Regulation of autophagy with rapamycin led to a clustering of patient's together with control's cells (Fig. 3E), matching previously reported effects.^50^ Modulating autophagy with chloroquine showed that a reduced colocalization of LC3 and LAMP1 led to a reduction TH+ neurons without altering the overall amount of neurons and increased the amount of GFAP levels (Fig. 3H,I). Treatment with different concentrations of HP‐β‐CD increased the presence of TFEB in the nuclei of patient‐derived neurons (Fig. 4A,B). Further studies need to be performed to establish the indirect link between TFEB nuclear translocation and the upregulation of *PINK1* independent mitophagy pathways. HP‐β‐CD treatment increased the amount of autophagy events as well as the colocalization of LAMP1‐p62 in TH+ neurons at concentrations above 5 μM, plateauing at 10 and 50 μM, concomitantly to the increase of TFEB nuclear translocation increase (Fig. 4C,D). Using cells edited with the Rosella construct tagging LC3, we observed similar effects (Fig. S7).

Differentially abundant protein analysis between untreated and treated patient organoids showed that the HP‐β‐CD has an effect not only in autophagy but also in proteins regulating dopaminergic neuronal differentiation (Fig. 4E,F). Levels of SOX9 and DLK1 were significantly reduced after treatment. SOX9 maintains the multipotency characteristics of neural precursor cells, reducing neuronal differentiation as well as increasing astrogliogenesis and astrocyte differentiation.^51^, ^52^, ^53^ Downregulation of DLK1 through paired like homeodomain 3 (PITX3) has been shown to be necessary for the differentiation of A9/substantia nigra pars compacta dopaminergic neurons.^54^, ^55^, ^56^ Levels of DLK1 increased over time in untreated patient organoids and were reduced after HP‐β‐CD treatment (Fig. 4F). Increased levels of RA51C, reported to help mtDNA replication and integrity, were seen after treatment. This coincided with reduced levels of IFNA1, known to reduce the autophagy clearance of mtDNA.^57^, ^58^ Reduced levels of LAMP2 in untreated patient organoids match previous reports in cerebrospinal fluid concentrations in patients with PD.^59^, ^60^ Similar results were reported in the brain extracts of patients with PD.^61^ LAMP2 is known to increase autophagic flux, reduce SNCA levels, and reduce the degeneration of dopaminergic neurons.^62^, ^63^ Treatment with HP‐β‐CD significantly increased the levels of LAMP2 in patient‐derived organoids (Fig. 4E,F). Moreover, HP‐β‐CD significantly reduced the levels of BIRC7, a protein known for inhibiting autophagy by reducing the levels of LC3II, autophagy related 5 (ATG5), and beclin 1 (BECN1).^64^ Levels of another autophagy‐related protein, 14‐3‐3ζ, were increased in untreated patient organoids (Fig. 4F). 14‐3‐3ζ negatively regulates the early stages of autophagy.^65^ Plus, 14‐3‐3 proteins are known regulators of the localization of TFEB between the nucleus and the cytoplasm, maintaining TFEB in the cytoplasm when it is phosphorylated.^66^ A significant reduction of 14‐3‐3ζ was observed after HP‐β‐CD treatment of patient organoids (Fig. 4F), suggesting that its downregulation facilitates the translocation of TFEB to the nucleus. Furthermore, treatment with HP‐β‐CD improved the impaired dopaminergic differentiation observed in the patient organoids (Fig. 4G,H). The induction of the translocation of TFEB is significant at concentrations of 5 and 10 μM (Fig. 4B), whereas the effect of HP‐β‐CD in the differentiation of the organoids was observed only at 5 μM. This would lead one to think that the beneficial aspects of the TFEB translocation when treating organoids with HP‐β‐CD for 30 consecutive days occur in a narrow concentration window.

Interestingly, it has been reported that increasing autophagy via expression of TFEB has positive effects in other neurodegenerative diseases, such as Alzheimer's disease, Niemann Pick disease, and Gaucher disease by improving the degradation of protein aggregates.^67^, ^68^, ^69^ Moreover, treatment with HP‐β‐CD is currently in a phase 2b/3 clinical trial for treating Niemann Pick disease (NPD, NCT02534844). Although the permeability of HP‐β‐CD to the blood–brain barrier is low (≈0.2% of blood circulating HP‐β‐CD)^70^, ^71^, ^72^ previous reports have shown a positive effect in reducing neurodegeneration in mice treated only systemically.^70^ We observed protection against the toxicity generated by MPTP after intraperitoneal treatment with HP‐β‐CD (Fig. 5). It has been reported that MPTP treatment increases brain‐barrier permeability,^73^ and this could have boosted the beneficial effects observed by HP‐β‐CD treatment. It is also possible that the pretreatment with HP‐β‐CD blocked MPTP‐induced dysfunction of the blood–brain barrier as it has been reported for other treatments.^74^

The present work demonstrates that HP‐β‐CD ameliorates the dopaminergic neuronal loss phenotype, confirming the therapeutic potential of modulating the autophagy/lysosomal pathway in the context of PD.

## Author Roles

(1) Research Project: A. Conception, B. Organization, C. Execution; (2) Statistical Analysis: A. Design, B. Execution, C. Review and Critique; (3) Manuscript: A. Writing of the First Draft, B. Review and Critique.

J.J.: 1A, 1B, 1C, 2A, 2B, 3A

K.B.: 1C

J.M.: 1B, 1C, 2A, 2B

C. Saraiva: 1B, 1C, 2A, 2B

S.S.‐S.: 1C, 2A, 2B

I.R.: 1B, 1C

A.G.: 1B, 1C, 2A, 2B

F.S.: 1B, 1C, 2A, 2B

G.Z.: 1B, 1C, 2A, 2B

L.M.S.: 1B, 1C, 3B

J.S.: 1C, 2A, 2B

J.A.‐F.: 1B, 1C

J.W.: 1B, 1C, 3B

G.G.‐G.: 1B, 1C

A.S.M.: 1B, 1C

X.Q.: 1B, 1C

A.V.: 1C, 2A, 2B

G.C.: 1B, 1C, 2A, 2B

I.B.: 1B

F.B.: 1C, 2A, 2B

C.J.: 1B, 1C

A.R.: 1B, 1C, 3B

W.L.: 1C, 2C

L.Y.: 1C, 2C

E.B.: 1B, 3B

G.A.: 1C, 2C, 3B

S.B.: 1B, 1C, 3B

R.S.: 1B, 1C, 2C

C. Schröder: 1B, 1C, 2C

P.M.A.A.: 1B, 1C, 3B

C.K.: 3B

R.K.: 3B

P.S.: 1B, 1C, 3B

J.C.S.: 1A, 2C, 3B

## Supporting information


**Figure S1**. Phenotyping pipeline. (**A**) Table summarizing the lines used in the article. (**B**) Time line and procedure for the generation of the lines and quality controls performed in the lines used. hiPSCs characterization scale bar = 100 μm. hNESCs characterization scale bar = 500 μm. Differentiation evaluation two‐dimensional scale bar = 100 μm. Differentiation evaluation three‐dimensional scale bar = 200 μm. (**C**) Representative images of a 14‐day differentiation neuronal culture on a chip of an OrganoPlate stained for tyrosine hydroxylase (TH), TUBB3, and a nuclear marker (scale bar = 100 μm). (**D**) Quantification of TH normalized to the neuronal area. Each dot represents a chip in an OrganoPlate over three replicates (chips control = 84, chips patient = 82). Statistical analysis was performed using Mann‐Whitney‐Wilcoxon test. *P < 0.05, **P < 0.01, ***P < 0.001. For panel **D,** all control and patient lines were used. TUBB3: neuron‐specific class III β‐tubulin.

**Figure S2**. Gene correction of *PINK1* mutations restores energetic profile and altered differentiation. (**A**) Representative images of a 14‐day differentiation neuronal two‐dimensional culture of controls, patients, and patients' gene‐corrected groups. RGB images of the markers tyrosine hydroxylase (TH), TUBB3, and Hoechst are presented with a zoomed region (scale bar = 100 μm). (**B**) Quantification of the markers TH, TUBB3, and Hoechst in a 14‐day differentiation neuronal two‐dimensional culture with their respective ratios and comparison between patient and gene‐corrected and control‐derived neurons. Images analyzed: fields of controls (fc) = 1868, fields gene corrected = 796, fields of patients (fp) = 416 were collected over three independent replicates using all control, patient, and patient gene corrected lines were used. (**C**) Representative scheme of the mitochondrial stress test profile for mitochondrial respiration and the areas used for the calculations obtained from the extracellular flux analysis. Representative oxygen consumption rates during the mitochondrial stress test. (**D**) Extracellular flux analysis (Seahorse) in neuroepithelial stem cells (NESCs) for evaluating mitochondrial respiratory capacity and efficiency between controls and patient‐derived and gene‐corrected cells. Data are pooled from three replicates. All control and patient lines were used; patient 1 GC lines was used. (**E**) Extracellular flux analysis (Seahorse) in NESCs for evaluating glycolytic activity. Data are pooled from three replicates. All control and patient lines were used; patient 1 GC lines was used. (**F**) Evaluation of spontaneous neuronal firing in a two‐dimensional culture by microelectrode measurements (MEA) represented by the mean firing rate, the number of spikes, and burst of the neuronal network between patient and gene‐corrected cells during differentiation. Data are pooled from three replicates. Patient line 1 and patient gene‐corrected lines were used. (**G**) Spike raster plots for single electrodes before and after treatment with the voltage‐dependent sodium channel blocker tetrodotoxin (TTX). Data are pooled from three replicates. Patient line 1 and patient gene‐corrected lines were used. (**H**) Representative raw data trace before and after addition of the voltage‐dependent sodium channel blocker TTX showing that the traces observed were produced by firing neurons. (**I**) Representative Western blot images of two patient‐derived *PINK1* p.Q456X‐mutant neurons (patient 1 and 2) and their respective isogenic gene‐corrected controls (patient 1 GC and patient 2 GC). hNESC were differentiated for 30 days in a two‐dimensional culture and treated overnight with 20 μM CCCP (or DMSO as vehicle). VDAC1 and Parkin protein levels were assessed by using the corresponding antibodies. Short and long exposure times allow the detection of unmodified and mono‐ubiquitinated forms of VDAC1, respectively. β‐actin was used as loading control. (**J**) Densitometric analysis of Western blot images presented in **E**. VDAC1 and Parkin signals were quantified by using Fiji. Statistical analyses for panels **B**, **D**, and **E** were performed using Kruskal‐Wallis and Dunn's tests for multiple comparisons. Adjustment of the *P*‐value for multiple tests was performed using Benjamini‐Hochberg. *P < 0.05, **P < 0.01, ***P < 0.001, ****P < 0.0001; ns, not significant. RGB: Red Green Blue; GC: gene corrected; hNESC: human neuroepithelial stem cells; CCCP: Carbonyl cyanide m‐chlorophenyl hydrazone; DMSO: Dimethyl sulfoxide; VDAC1: Voltage‐dependent anion‐selective channel 1. TUBB3: neuron‐specific class III β‐tubulin.

**Figure S3**. Mapping of significantly enriched KEGG pathways on the protein–protein interaction network between control and patient‐derived organoids at day 30 of neuronal differentiation. (**A**–**D**) Mapping of significantly enriched KEGG pathways on the protein–protein interaction network. KEGG pathways were tested for enrichment in proteins present in the network compared to the human genome and were considered significantly enriched if their *P*‐value adjusted by Benjamini‐Hochberg was <0.05. Nodes corresponding to proteins that belong to a selection of significantly enriched pathways are highlighted with different colors on the STRING network. The border of the nodes depicts the log fold change (logFC) of the pathway proteins in the comparison of control and patient‐derived organoids. Control line 1 and patient line 1 were used for the proteomics experiments. KEGG: (Kyoto Encyclopedia of Genes and Genomes).

**Figure S4**. Differential abundance of proteins between control and patient‐derived organoids at day 10 of neuronal differentiation. (**A**) Volcano plot of proteomics data. The *x* axis represents the log fold change (logFC) between patient‐derived and control organoids, with positive logFC indicating that the protein is more abundant in patient data than in control data, and the opposite for negative logFC. The *y* axis represents the *P*‐value of the comparison adjusted for multiple testing using Benjamini‐Hochberg. Proteins with adjusted *P*‐value <0.05 and absolute logFC >0.5 were considered differential. (**B**) Network of the protein–protein interactions among the differential proteins obtained from the STRING database. Interactions obtained from all data sources and with a confidence score >0.9 (high) were considered. Differential proteins that are not reported to interact with other differential proteins are not represented. (**C**–**I**) Mapping of significantly enriched KEGG pathways on the protein–protein interaction network. KEGG pathways were tested for enrichment in proteins present in the network compared to the human genome and were considered significantly enriched if their *P*‐value adjusted by Benjamini‐Hochberg was <0.05. Nodes corresponding to proteins that belong to a selection of significantly enriched pathways are highlighted with different colors on the STRING network. The border of the nodes depicts the logFC of the pathway proteins in the comparison of control and patient‐derived organoids. Control line 1 and patient line 1 were used for the proteomics experiments. KEGG: (Kyoto Encyclopedia of Genes and Genomes).

**Figure S5**. Differential abundance of proteins between control and patient‐derived organoids at day 20 of neuronal differentiation. (**A**) Volcano plot of proteomics data. The *x* axis represents the log fold change (logFC) between patient‐derived and control organoids, with positive logFC indicating that the protein is more abundant in patient data than in control data, and the opposite for negative logFC. The *y* axis represents the *P*‐value of the comparison adjusted for multiple testing using Benjamini‐Hochberg. Proteins with adjusted *P*‐value <0.05 and absolute logFC >0.5 were considered differential. (**B**) Network of the protein–protein interactions among the differential proteins obtained from the STRING database. Interactions obtained from all data sources and with a confidence score > 0.9 (high) were considered. Differential proteins that are not reported to interact with other differential proteins are not represented. (**C**–**K**) Mapping of significantly enriched KEGG pathways on the protein–protein interaction network. KEGG pathways were tested for enrichment in proteins present in the network compared to the human genome and were considered significantly enriched if their *P*‐value adjusted by Benjamini‐Hochberg was <0.05. Nodes corresponding to proteins that belong to a selection of significantly enriched pathways are highlighted with different colors on the STRING network. The border of the nodes depicts the logFC of the pathway proteins in the comparison of control and patient‐derived organoids. Control line 1 and patient line 1 were used for the proteomics experiments. KEGG: (Kyoto Encyclopedia of Genes and Genomes).

**Figure S6**. Autophagy levels and effect of chloroquine treatment. (**A**) Western blot of β‐actin and LC3 of differentiated neurons in a two‐dimensional culture. (**B**) Quantification of the LC3II to LC3I ratio normalized to β‐actin levels. (**C**) Time series quantification of the mitophagy total area during neuronal differentiation for 14 days in a two‐dimensional culture. Measurements were performed once a day during the entire differentiation protocol. Images analyzed: fields of controls (fc) = 97–219 and fields of patients (fp) =126–224 range measured per day for 14 days. Acquisition was performed at 60× sampling randomly 15 fields per well. Five wells of control 1 and 5 wells of patient 3 were acquired per replicate over three independent replicates. (**D**) Representative images of the effect of the rapamycin treatment in the flux of autophagy in control line 1 and patient line 3 hiPSCs. (**E**) Quantification of immunostaining for LC3, lysosomal associated membrane protein 1 (LAMP1), and tyrosine hydroxylase positive (TH+) and their respective colocalizations, normalized to nuclear area at different chloroquine concentrations. (**F**) Quantification of immunostaining for glial fibrillary acidic protein (GFAP), TUBB3, and TH+ and their respective colocalizations, normalized to nuclear area at different chloroquine concentrations. Except for panels **C** and **D**, all control and patient lines were used. Statistical analysis for panel **B** was performed with Mann–Whitney's test. For the rest of the panels, statistical analysis was performed using Kruskal–Wallis and Dunn's tests for multiple comparisons. Adjustment of the *P*‐value for multiple tests was performed using Benjamini‐Hochberg. *P < 0.05, **P < 0.01, ***P < 0.001, ****P < 0.0001; ns, not significant. LC3: Microtubule‐associated proteins 1A/1B light chain 3B; TUBB3: neuron‐specific class III β‐tubulin.

**Figure S7** The 2‐hydroxypropyl‐β‐cyclodextrin (HP‐β‐CD) treatment improves impaired autophagy. (**A**) Relative quantification of 14 days two‐dimensional neurons' gene expression of *FKBP8* and *FUNDC1* against housekeeping gene (*TBP*) in treated and untreated conditions over three independent replicates. (**B**) Time series quantification and comparison of the mitophagy capacity during neuronal differentiation for 14 days in a two‐dimensional culture between untreated and HP‐β‐CD‐treated control and patient‐derived neurons. Different properties of mitochondria and mitophagy events were assessed. Measurements were performed once a day during the entire differentiation protocol. Images acquired: fields control treated (fc) = 157–225 and fields patient treated (fp) = 215–225 were obtained per day for 14 days per replicate over three independent replicates. Control 1 and patient 3 lines were used. The values of control and patients untreated are the same as in Fig. 3D, added here for visual comparison. Statistical analysis was performed comparing only patient untreated versus patient + HP‐β‐CD. (**C**) Quantification of the autophagy events at day 14 of differentiation of neurons in a two‐dimensional culture tagged with the Rosella construct labeling LC3, with different HP‐β‐CD treatment concentrations. (**D**) For each protein cluster, the average scaled expression throughout the differentiation is reported for each condition separately. This serves to represent the expression pattern followed by the proteins in each cluster across the different conditions. Control line 1 and patient line 1 were used for the proteomics experiments. For panel **A**, all control and patient lines were used. For panel **C**, control line 2, control line 3, and all patient lines were used. For panel **B,** statistical analysis was performed using a nonparametric test for repeated measures in factorial design (nparLD). For the remainder of the panels, statistical analysis was performed using Kruskal–Wallis and Dunn's tests for multiple comparisons. Adjustment of the *P*‐value for multiple tests was performed using Benjamini‐Hochberg (BH).

**Figure S8**. Organoid Image analysis and further compound testing. (**A**) Representative images of organoid sections with the respective masks identifying tyrosine hydroxylase (TH), TUBB3, and Hoechst. Scale bar = 200 μm. (**B**) Quantification of the markers TH, TUBB3, and Hoechst in a 14 day differentiation neuronal culture with their respective ratios and comparison between untreated and 1 μM 2‐hydroxypropyl‐β‐cyclodextrin (HP‐β‐CD)–treated *PRKN* patient‐derived neurons. For panel **B**, all control lines and the patient parkin line were used. Statistical analysis for panel **B** was performed using Kruskal–Wallis and Dunn's tests for multiple comparisons. TUBB3: neuron‐specific class III β‐tubulin.

**Figure S9**. The 2‐hydroxypropyl‐β‐cyclodextrin (HP‐β‐CD) mixture. Adjustment of the *P*‐value for multiple tests was performed using Benjamini‐Hochberg. *P < 0.05, **P < 0.01, ***P < 0.001, ****P < 0.0001; ns, not significant.

**Table S1**. Differentially expressed proteins of Patient versus Control derived organoids at day 10 of differentiation in untreated conditions.

**Table S2**. Differentially expressed proteins of Patient versus Control derived organoids at day 10 of differentiation in untreated conditions.

**Table S3**. Differentially expressed proteins of Patient versus Control derived organoids at day 10 of differentiation in untreated conditions.

**Table S4**. Differentially expressed proteins of untreated Patient versus treated patients derived organoids at day 10 of differentiation in untreated conditions.

**Table S5**. Differentially expressed proteins of untreated Patient versus treated patients derived organoids at day 20 of differentiation in untreated conditions.

**Table S6**. Differentially expressed proteins of untreated Patient versus treated patients derived organoids at day 30 of differentiation in untreated conditions.

**Table S7**. Detailed information about the reagents and resources used in the paper.

**Table S8**. Medians of the differentiation experiments.

## Data Availability

All original and processed data including the scripts used in this work are publicly available at this doi: https://doi.org/10.17881/c80y-2k58
