# Supplementary material for: Parkinson's Disease Phenotypes in Patient Neuronal Cultures and Brain Organoids Improved by 2‐Hydroxypropyl‐β‐Cyclodextrin Treatment
Source: Mov Disord. 2021 Oct 12;37(1):80–94. doi: 10.1002/mds.28810 (PMC9291890; doi:10.1002/mds.28810)

Fig. S1

A

	Mutation	Gender	Age onset	Age sampling	Source	Database
Healthy control 1	-	♀	-	81	Reinhardt et al., 2013	506
Healthy control 2	-	♀	-	53	Reinhardt et al., 2013	509
Healthy control 3	-	♂	-	59	W. Skarnes	548
Patient PINK1 1	Q456X	♀	61	69	C. Klein	537
Patient PINK1 2	Q456X	♀	53	61	C. Klein	551
Patient PINK1 3	I368N	♂	33	64	Coriell	511
Patient Parkin	R275W	♀	43	61	Coriell	513
Patient PINK1 1 GC	-	♀	61	69	C. Klein	571
Patient PINK1 2 GC	-	♀	53	61	C. Klein	569
Patient PINK1 3 GC	-	♂	33	64	Coriell	572

B

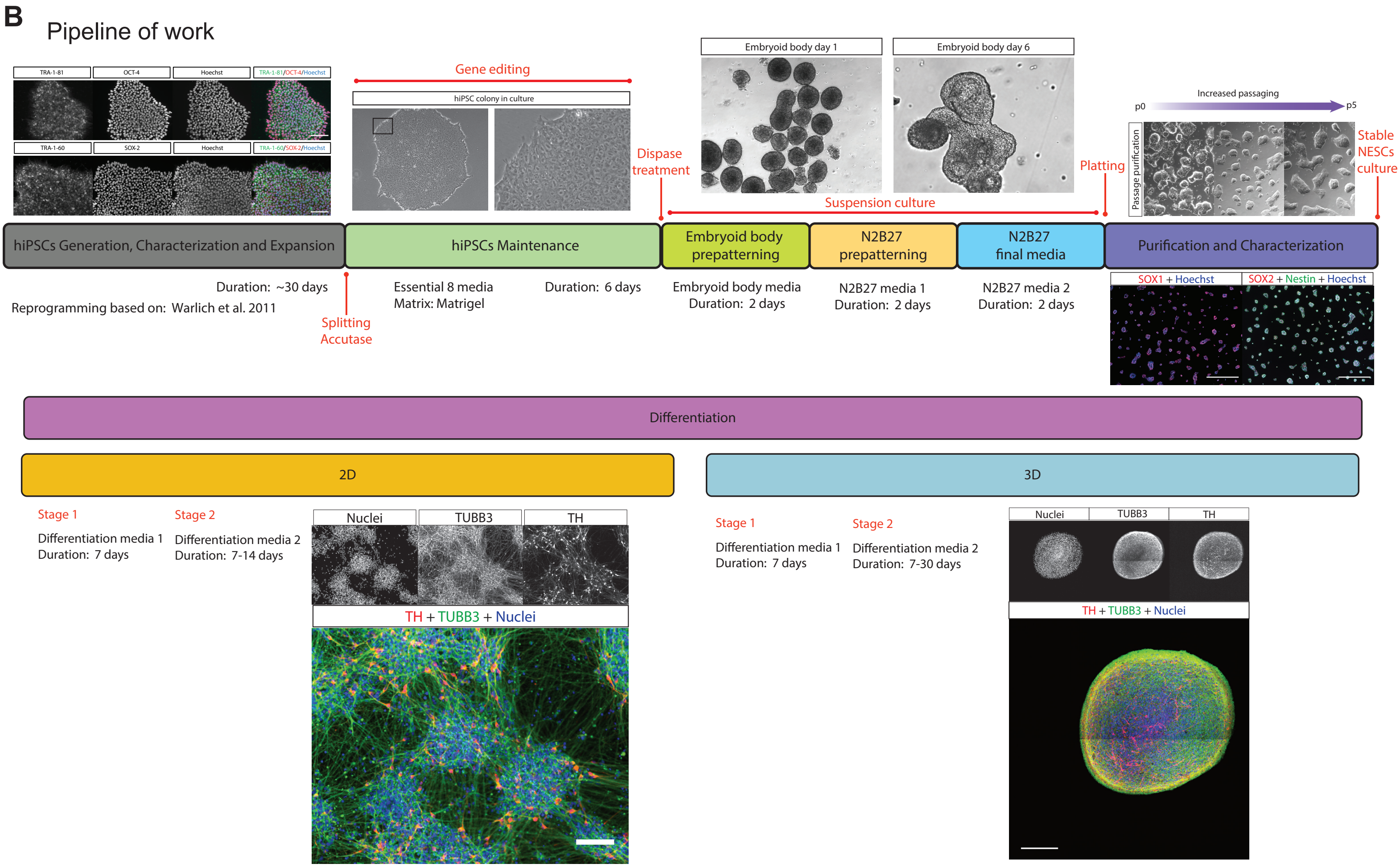

C

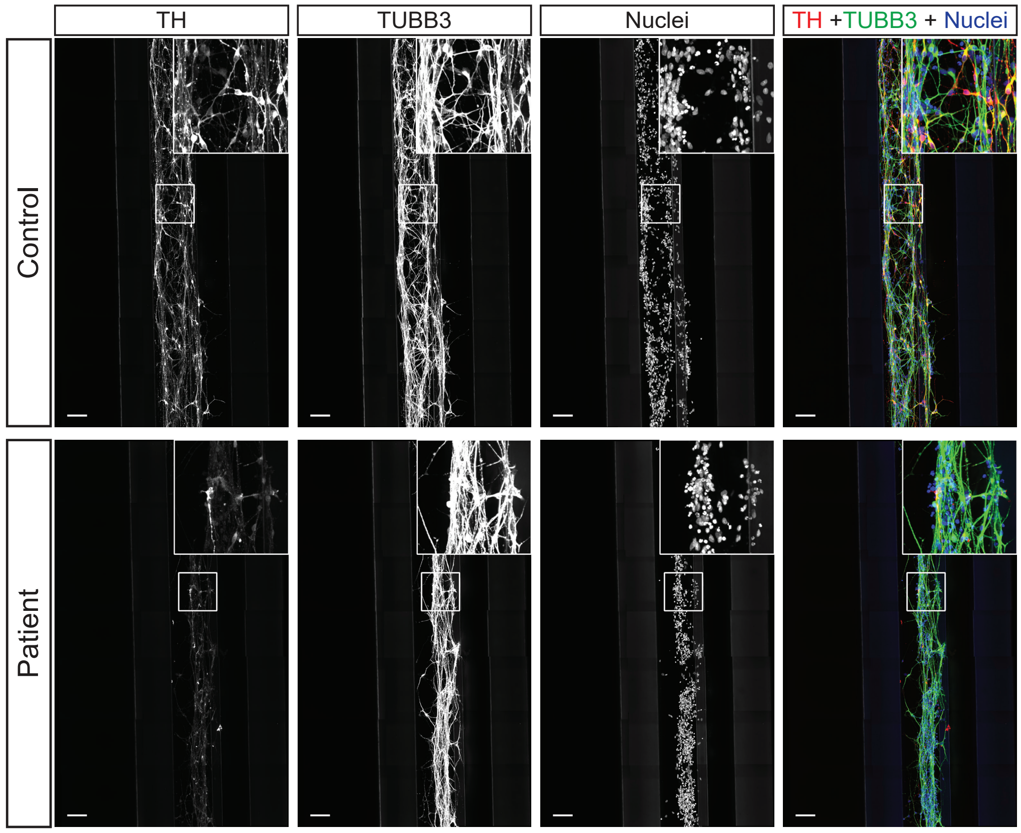

D

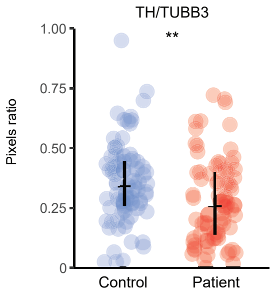

Supplement: Supplementary file 1 — Figure S1. Phenotyping pipeline. (A) Table summarizing the lines used in the article. (B) Time line and procedure for the generation of the lines and quality controls performed in the lines used. hiPSCs characterization scale bar = 100 μm. hNESCs characterization scale bar = 500 μm. Differentiation evaluation two‐dimensional scale bar = 100 μm. Differentiation evaluation three‐dimensional scale bar = 200 μm. (C) Representative images of a 14‐day differentiation neuronal culture on a chip of an OrganoPlate stained for tyrosine hydroxylase (TH), TUBB3, and a nuclear marker (scale bar = 100 μm). (D) Quantification of TH normalized to the neuronal area. Each dot represents a chip in an OrganoPlate over three replicates (chips control = 84, chips patient = 82). Statistical analysis was performed using Mann‐Whitney‐Wilcoxon test. *P < 0.05, **P < 0.01, ***P < 0.001. For panel D, all control and patient lines were used. TUBB3: neuron‐specific class III β‐tubulin. [file MDS-37-80-s004.pdf]
