# Supplementary figures and images for: Parkinson's Disease Phenotypes in Patient Neuronal Cultures and Brain Organoids Improved by 2‐Hydroxypropyl‐β‐Cyclodextrin Treatment
Source: Mov Disord. 2021 Oct 12;37(1):80–94. doi: 10.1002/mds.28810 (PMC9291890; doi:10.1002/mds.28810)

**Fig. S2**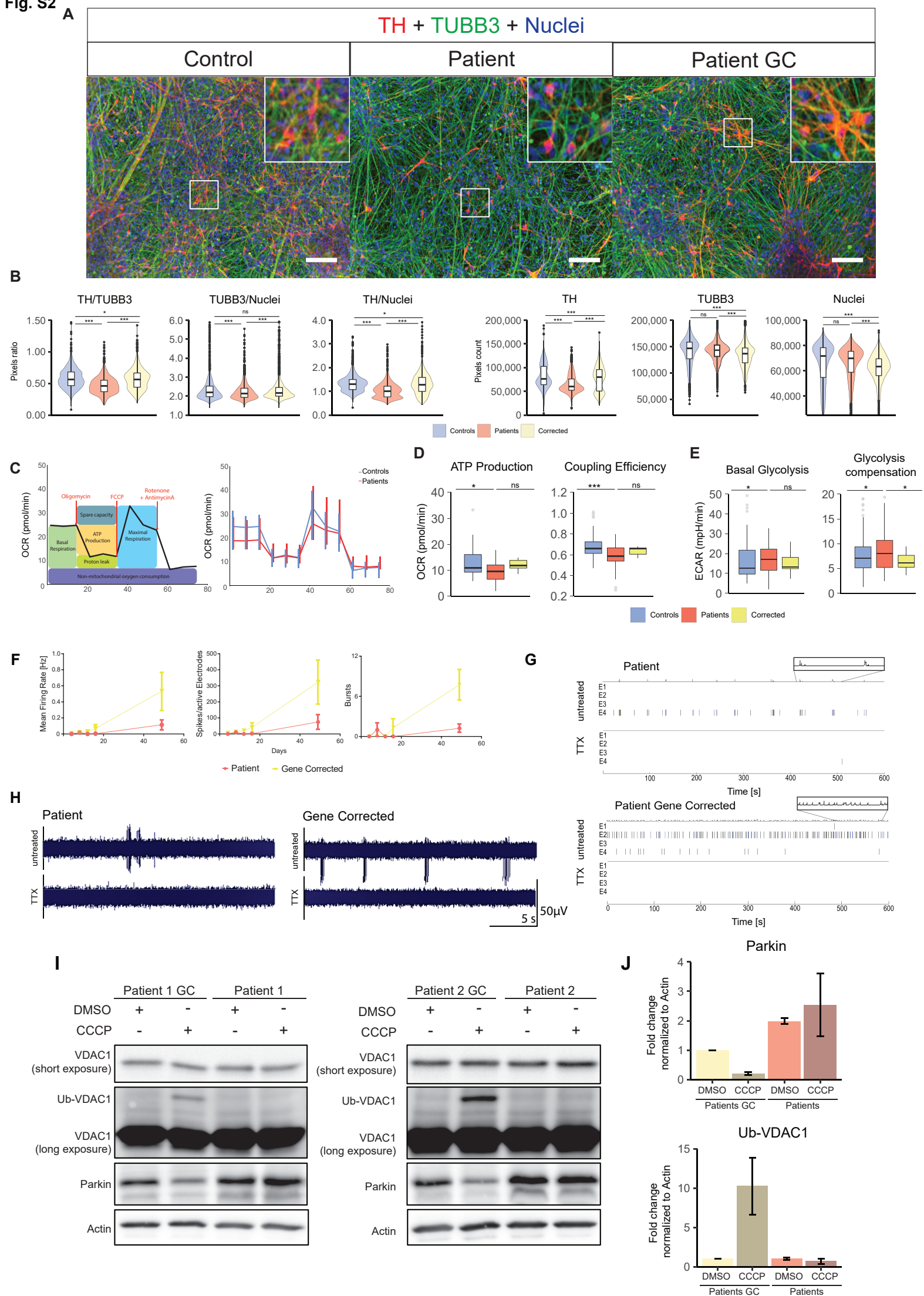

Supplement: Supplementary file 2 — Figure S2. Gene correction of PINK1 mutations restores energetic profile and altered differentiation. (A) Representative images of a 14‐day differentiation neuronal two‐dimensional culture of controls, patients, and patients' gene‐corrected groups. RGB images of the markers tyrosine hydroxylase (TH), TUBB3, and Hoechst are presented with a zoomed region (scale bar = 100 μm). (B) Quantification of the markers TH, TUBB3, and Hoechst in a 14‐day differentiation neuronal two‐dimensional culture with their respective ratios and comparison between patient and gene‐corrected and control‐derived neurons. Images analyzed: fields of controls (fc) = 1868, fields gene corrected = 796, fields of patients (fp) = 416 were collected over three independent replicates using all control, patient, and patient gene corrected lines were used. (C) Representative scheme of the mitochondrial stress test profile for mitochondrial respiration and the areas used for the calculations obtained from the extracellular flux analysis. Representative oxygen consumption rates during the mitochondrial stress test. (D) Extracellular flux analysis (Seahorse) in neuroepithelial stem cells (NESCs) for evaluating mitochondrial respiratory capacity and efficiency between controls and patient‐derived and gene‐corrected cells. Data are pooled from three replicates. All control and patient lines were used; patient 1 GC lines was used. (E) Extracellular flux analysis (Seahorse) in NESCs for evaluating glycolytic activity. Data are pooled from three replicates. All control and patient lines were used; patient 1 GC lines was used. (F) Evaluation of spontaneous neuronal firing in a two‐dimensional culture by microelectrode measurements (MEA) represented by the mean firing rate, the number of spikes, and burst of the neuronal network between patient and gene‐corrected cells during differentiation. Data are pooled from three replicates. Patient line 1 and patient gene‐corrected lines were used. (G) Spike raster plots [file MDS-37-80-s015.pdf]

Fig. S3

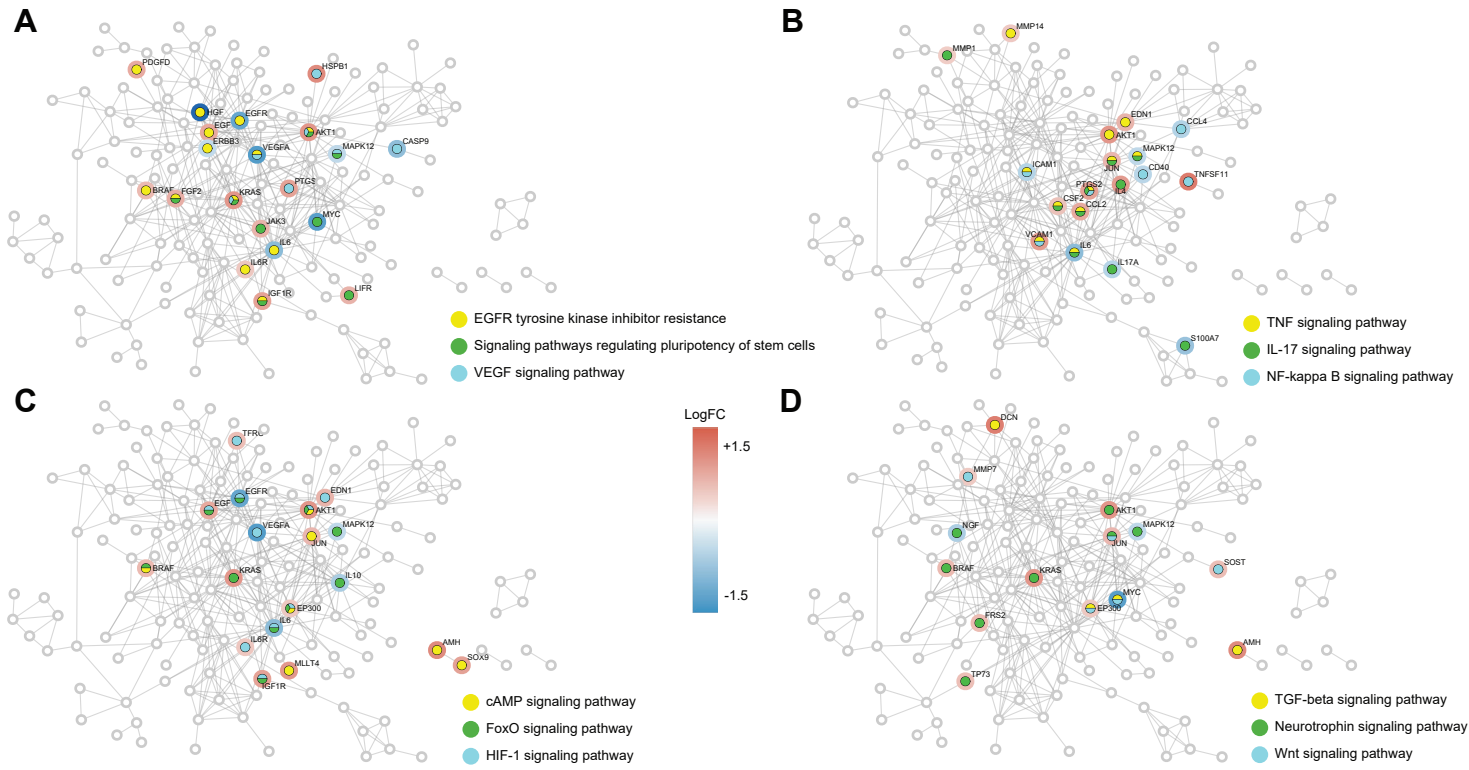

Supplement: Supplementary file 3 — Figure S3. Mapping of significantly enriched KEGG pathways on the protein–protein interaction network between control and patient‐derived organoids at day 30 of neuronal differentiation. (A–D) Mapping of significantly enriched KEGG pathways on the protein–protein interaction network. KEGG pathways were tested for enrichment in proteins present in the network compared to the human genome and were considered significantly enriched if their P‐value adjusted by Benjamini‐Hochberg was <0.05. Nodes corresponding to proteins that belong to a selection of significantly enriched pathways are highlighted with different colors on the STRING network. The border of the nodes depicts the log fold change (logFC) of the pathway proteins in the comparison of control and patient‐derived organoids. Control line 1 and patient line 1 were used for the proteomics experiments. KEGG: (Kyoto Encyclopedia of Genes and Genomes). [file MDS-37-80-s013.pdf]

**Fig. S4**

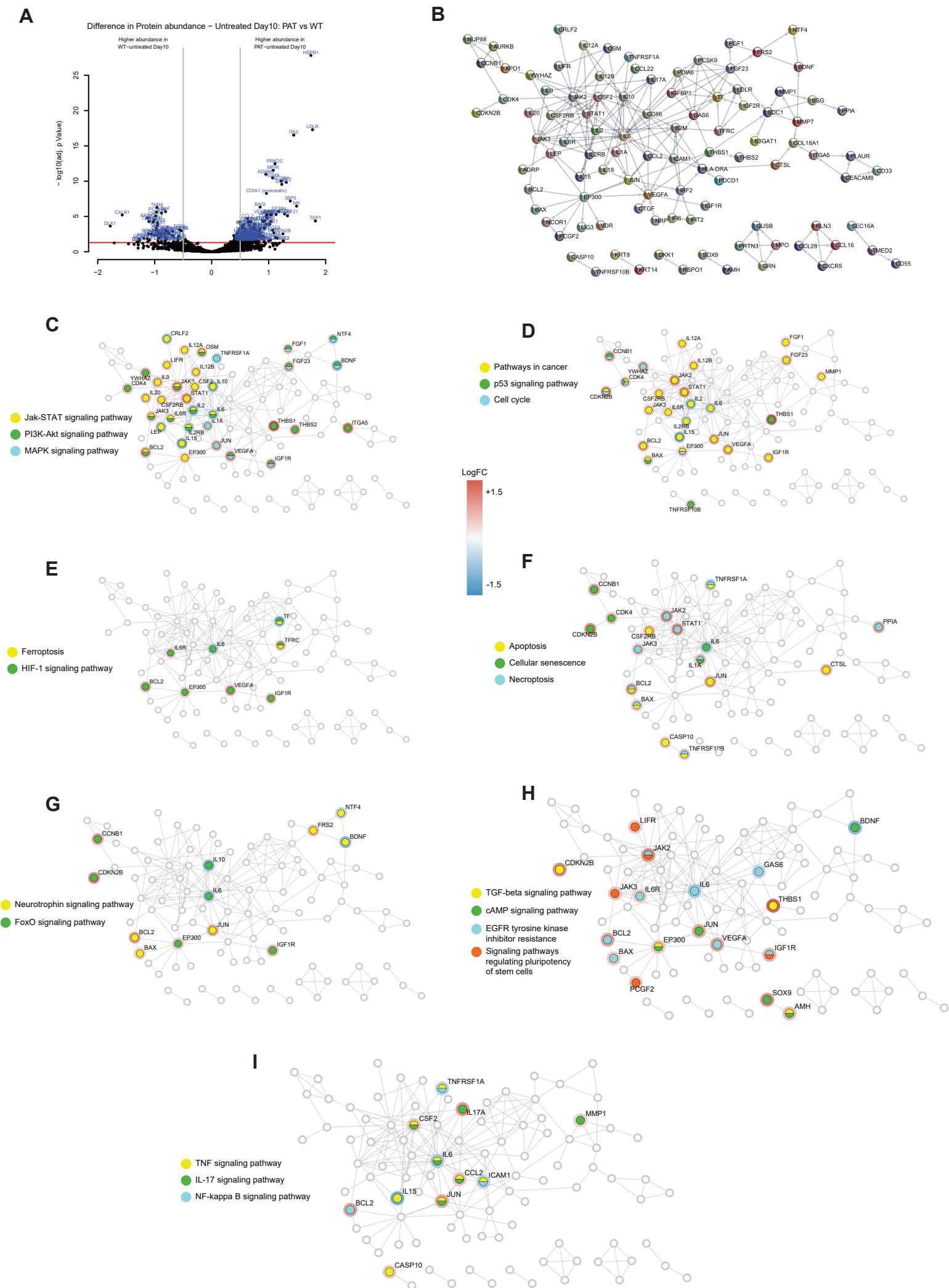

Supplement: Supplementary file 4 — Figure S4. Differential abundance of proteins between control and patient‐derived organoids at day 10 of neuronal differentiation. (A) Volcano plot of proteomics data. The x axis represents the log fold change (logFC) between patient‐derived and control organoids, with positive logFC indicating that the protein is more abundant in patient data than in control data, and the opposite for negative logFC. The y axis represents the P‐value of the comparison adjusted for multiple testing using Benjamini‐Hochberg. Proteins with adjusted P‐value <0.05 and absolute logFC >0.5 were considered differential. (B) Network of the protein–protein interactions among the differential proteins obtained from the STRING database. Interactions obtained from all data sources and with a confidence score >0.9 (high) were considered. Differential proteins that are not reported to interact with other differential proteins are not represented. (C–I) Mapping of significantly enriched KEGG pathways on the protein–protein interaction network. KEGG pathways were tested for enrichment in proteins present in the network compared to the human genome and were considered significantly enriched if their P‐value adjusted by Benjamini‐Hochberg was <0.05. Nodes corresponding to proteins that belong to a selection of significantly enriched pathways are highlighted with different colors on the STRING network. The border of the nodes depicts the logFC of the pathway proteins in the comparison of control and patient‐derived organoids. Control line 1 and patient line 1 were used for the proteomics experiments. KEGG: (Kyoto Encyclopedia of Genes and Genomes). [file MDS-37-80-s016.pdf]

Fig. S5

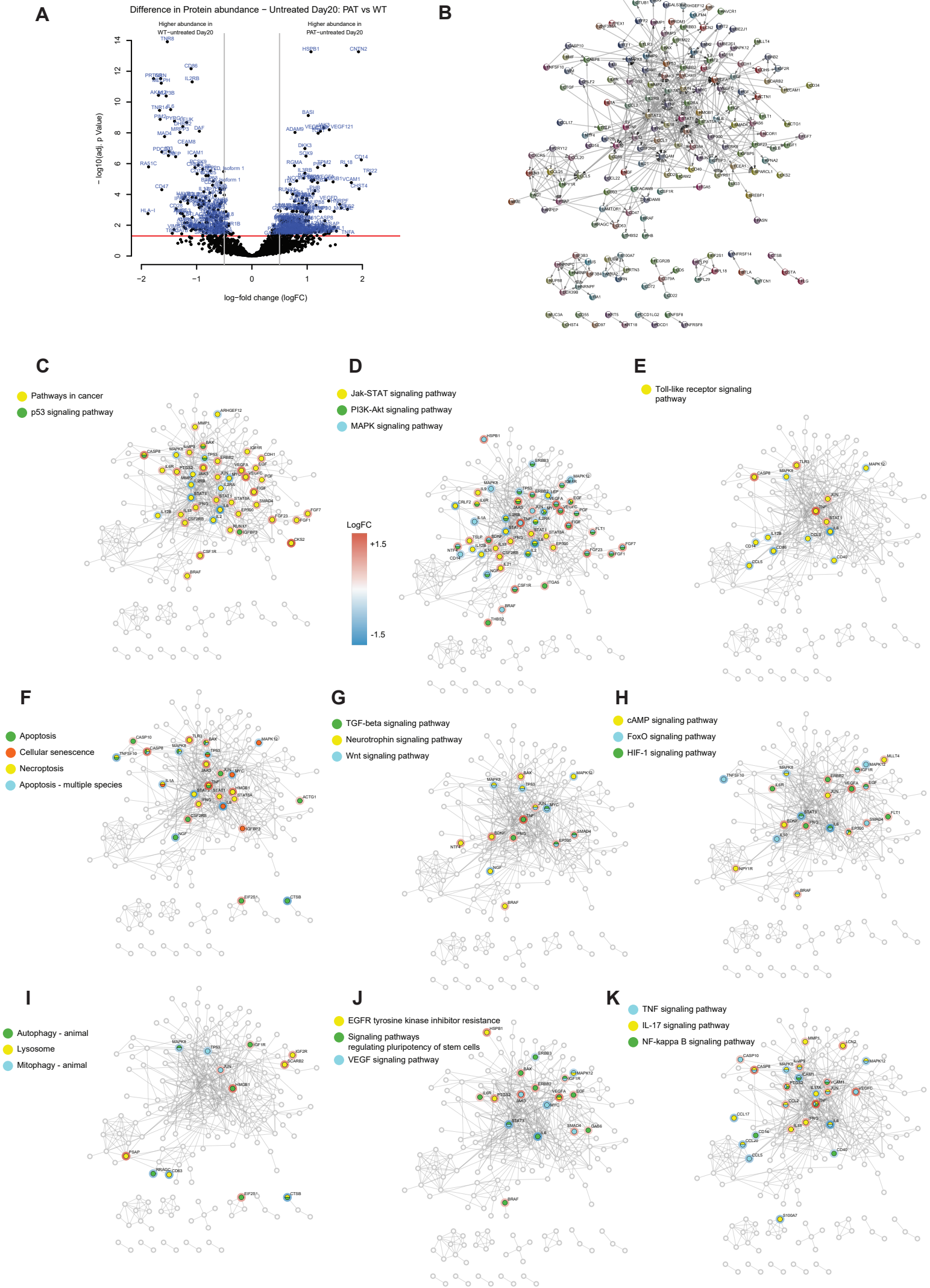

Supplement: Supplementary file 5 — Figure S5. Differential abundance of proteins between control and patient‐derived organoids at day 20 of neuronal differentiation. (A) Volcano plot of proteomics data. The x axis represents the log fold change (logFC) between patient‐derived and control organoids, with positive logFC indicating that the protein is more abundant in patient data than in control data, and the opposite for negative logFC. The y axis represents the P‐value of the comparison adjusted for multiple testing using Benjamini‐Hochberg. Proteins with adjusted P‐value <0.05 and absolute logFC >0.5 were considered differential. (B) Network of the protein–protein interactions among the differential proteins obtained from the STRING database. Interactions obtained from all data sources and with a confidence score > 0.9 (high) were considered. Differential proteins that are not reported to interact with other differential proteins are not represented. (C–K) Mapping of significantly enriched KEGG pathways on the protein–protein interaction network. KEGG pathways were tested for enrichment in proteins present in the network compared to the human genome and were considered significantly enriched if their P‐value adjusted by Benjamini‐Hochberg was <0.05. Nodes corresponding to proteins that belong to a selection of significantly enriched pathways are highlighted with different colors on the STRING network. The border of the nodes depicts the logFC of the pathway proteins in the comparison of control and patient‐derived organoids. Control line 1 and patient line 1 were used for the proteomics experiments. KEGG: (Kyoto Encyclopedia of Genes and Genomes). [file MDS-37-80-s006.pdf]

Fig. S6

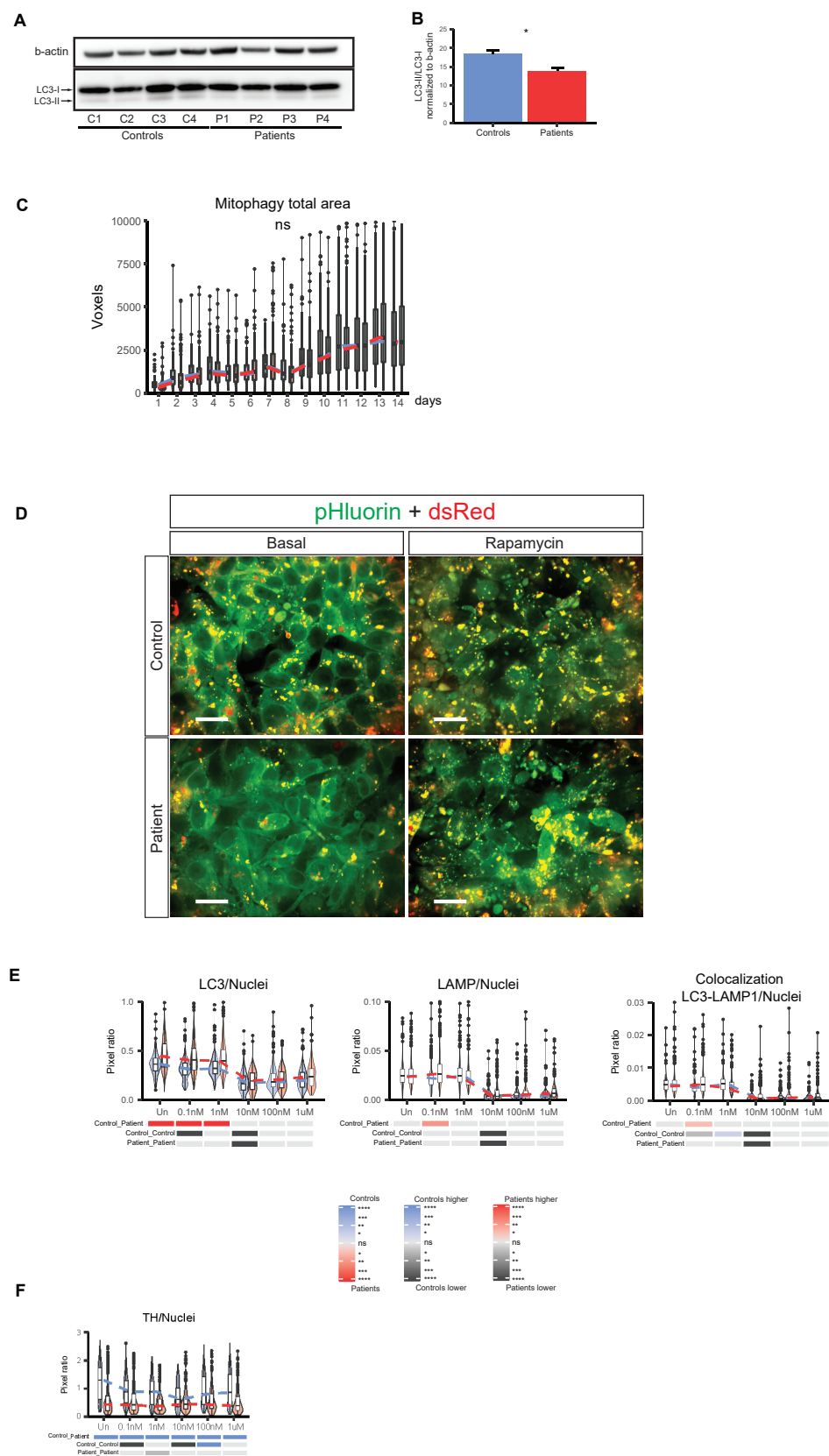

Supplement: Supplementary file 6 — Figure S6. Autophagy levels and effect of chloroquine treatment. (A) Western blot of β‐actin and LC3 of differentiated neurons in a two‐dimensional culture. (B) Quantification of the LC3II to LC3I ratio normalized to β‐actin levels. (C) Time series quantification of the mitophagy total area during neuronal differentiation for 14 days in a two‐dimensional culture. Measurements were performed once a day during the entire differentiation protocol. Images analyzed: fields of controls (fc) = 97–219 and fields of patients (fp) =126–224 range measured per day for 14 days. Acquisition was performed at 60× sampling randomly 15 fields per well. Five wells of control 1 and 5 wells of patient 3 were acquired per replicate over three independent replicates. (D) Representative images of the effect of the rapamycin treatment in the flux of autophagy in control line 1 and patient line 3 hiPSCs. (E) Quantification of immunostaining for LC3, lysosomal associated membrane protein 1 (LAMP1), and tyrosine hydroxylase positive (TH+) and their respective colocalizations, normalized to nuclear area at different chloroquine concentrations. (F) Quantification of immunostaining for glial fibrillary acidic protein (GFAP), TUBB3, and TH+ and their respective colocalizations, normalized to nuclear area at different chloroquine concentrations. Except for panels C and D, all control and patient lines were used. Statistical analysis for panel B was performed with Mann–Whitney's test. For the rest of the panels, statistical analysis was performed using Kruskal–Wallis and Dunn's tests for multiple comparisons. Adjustment of the P‐value for multiple tests was performed using Benjamini‐Hochberg. *P < 0.05, **P < 0.01, ***P < 0.001, ****P < 0.0001; ns, not significant. LC3: Microtubule‐associated proteins 1A/1B light chain 3B; TUBB3: neuron‐specific class III β‐tubulin. [file MDS-37-80-s007.pdf]

Fig. S7

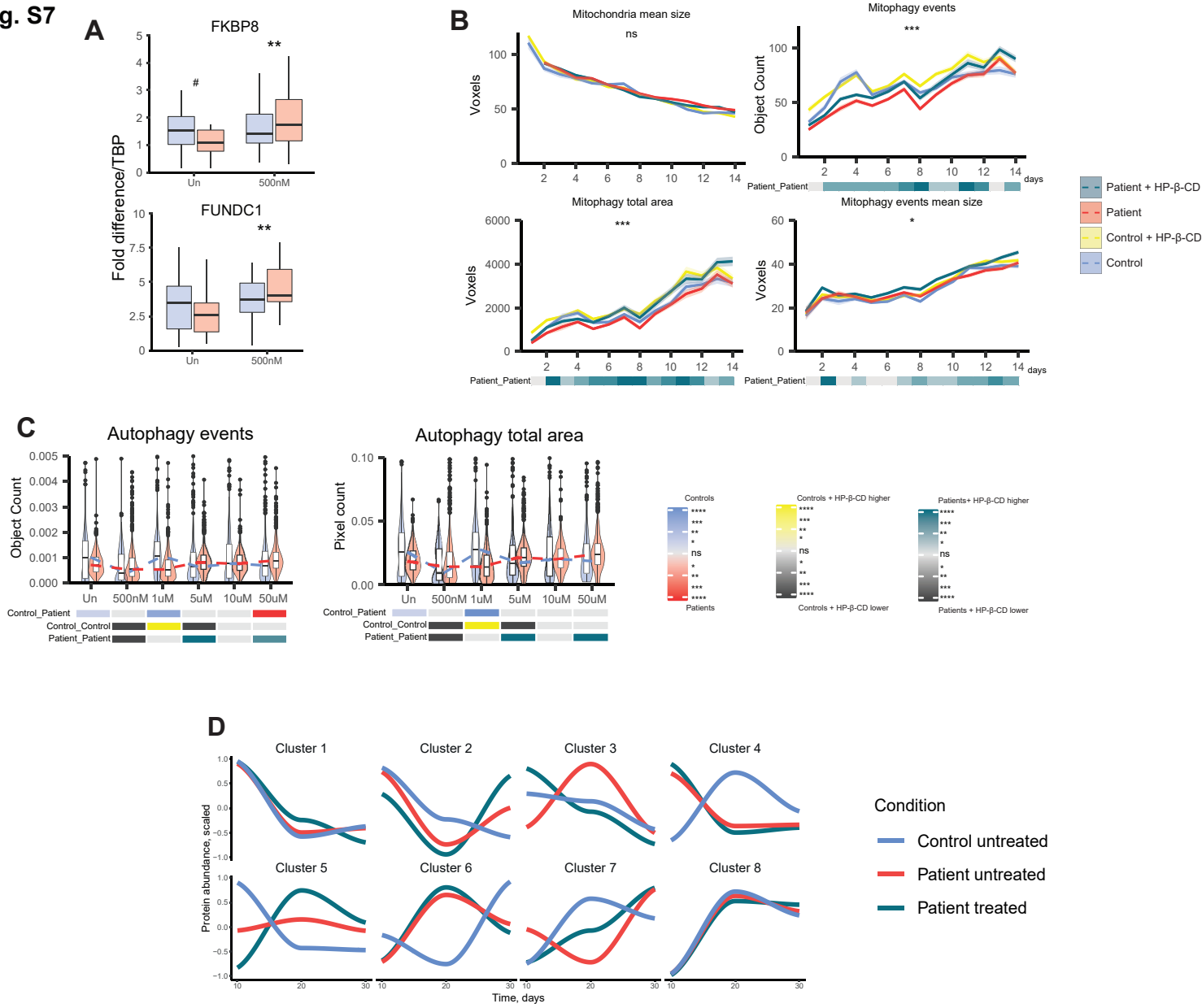

Supplement: Supplementary file 7 — Figure S7 The 2‐hydroxypropyl‐β‐cyclodextrin (HP‐β‐CD) treatment improves impaired autophagy. (A) Relative quantification of 14 days two‐dimensional neurons' gene expression of FKBP8 and FUNDC1 against housekeeping gene (TBP) in treated and untreated conditions over three independent replicates. (B) Time series quantification and comparison of the mitophagy capacity during neuronal differentiation for 14 days in a two‐dimensional culture between untreated and HP‐β‐CD‐treated control and patient‐derived neurons. Different properties of mitochondria and mitophagy events were assessed. Measurements were performed once a day during the entire differentiation protocol. Images acquired: fields control treated (fc) = 157–225 and fields patient treated (fp) = 215–225 were obtained per day for 14 days per replicate over three independent replicates. Control 1 and patient 3 lines were used. The values of control and patients untreated are the same as in Fig. 3D, added here for visual comparison. Statistical analysis was performed comparing only patient untreated versus patient + HP‐β‐CD. (C) Quantification of the autophagy events at day 14 of differentiation of neurons in a two‐dimensional culture tagged with the Rosella construct labeling LC3, with different HP‐β‐CD treatment concentrations. (D) For each protein cluster, the average scaled expression throughout the differentiation is reported for each condition separately. This serves to represent the expression pattern followed by the proteins in each cluster across the different conditions. Control line 1 and patient line 1 were used for the proteomics experiments. For panel A, all control and patient lines were used. For panel C, control line 2, control line 3, and all patient lines were used. For panel B, statistical analysis was performed using a nonparametric test for repeated measures in factorial design (nparLD). For the remainder of the panels, statistical analysis was performed using Kruskal–Wallis and Dunn's tests [file MDS-37-80-s017.pdf]

Fig. S8

A

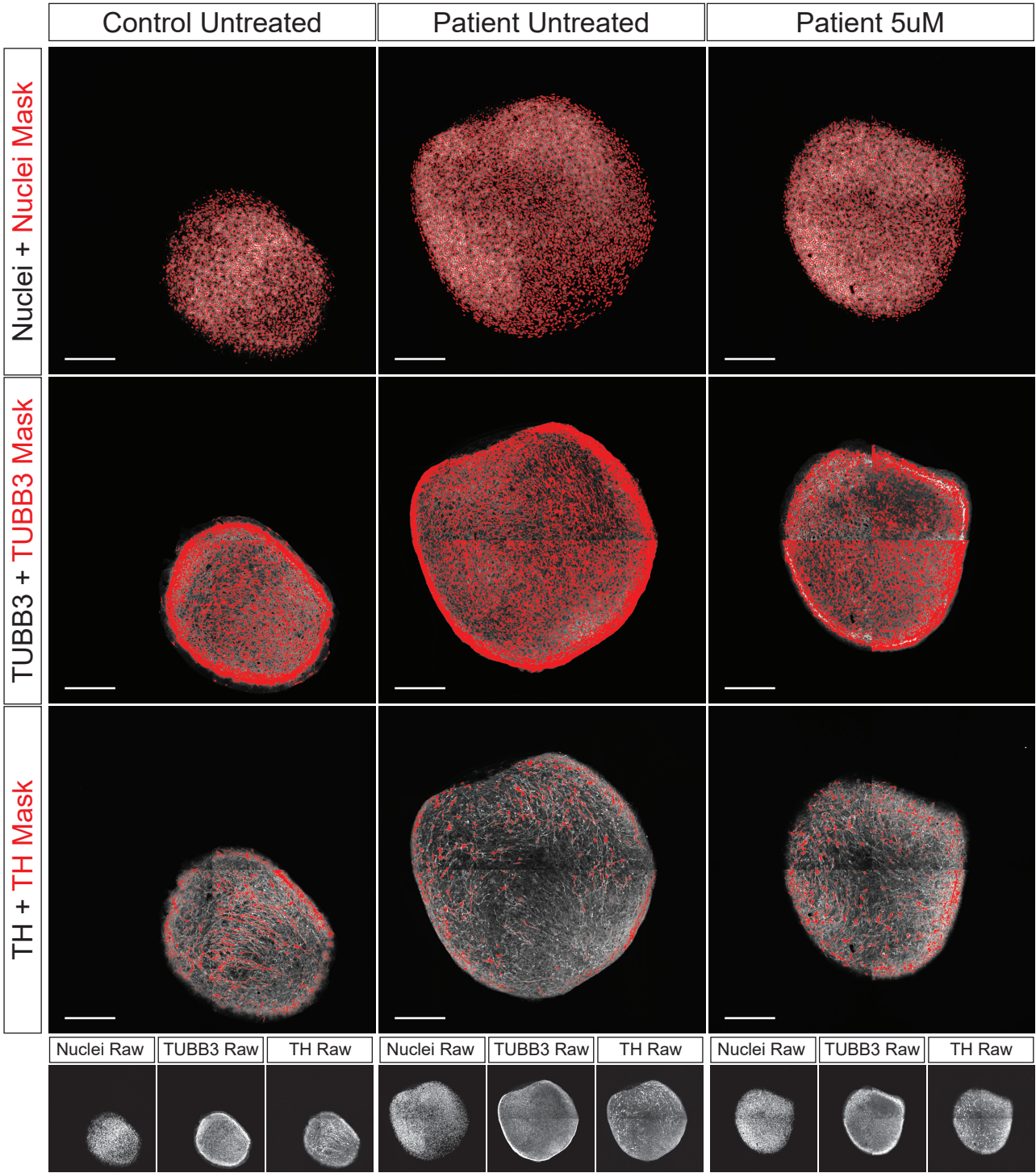

B

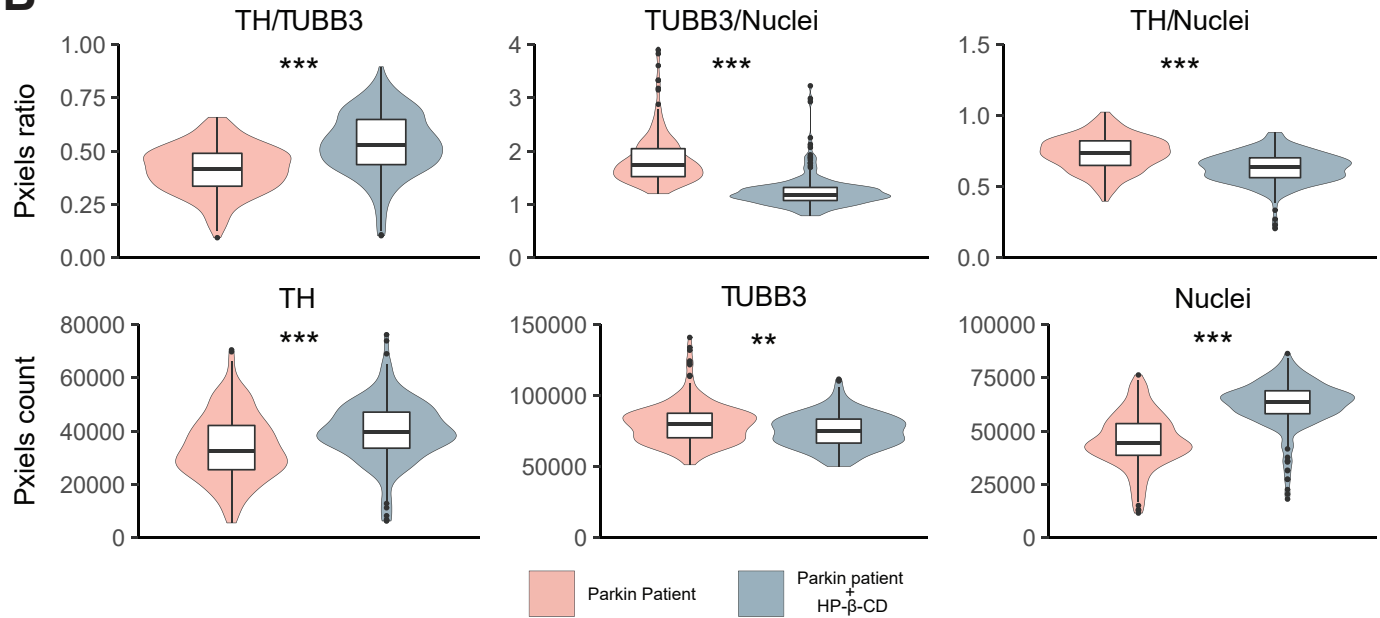

Supplement: Supplementary file 8 — Figure S8. Organoid Image analysis and further compound testing. (A) Representative images of organoid sections with the respective masks identifying tyrosine hydroxylase (TH), TUBB3, and Hoechst. Scale bar = 200 μm. (B) Quantification of the markers TH, TUBB3, and Hoechst in a 14 day differentiation neuronal culture with their respective ratios and comparison between untreated and 1 μM 2‐hydroxypropyl‐β‐cyclodextrin (HP‐β‐CD)–treated PRKN patient‐derived neurons. For panel B, all control lines and the patient parkin line were used. Statistical analysis for panel B was performed using Kruskal–Wallis and Dunn's tests for multiple comparisons. TUBB3: neuron‐specific class III β‐tubulin. [file MDS-37-80-s011.pdf]

Fig. S9

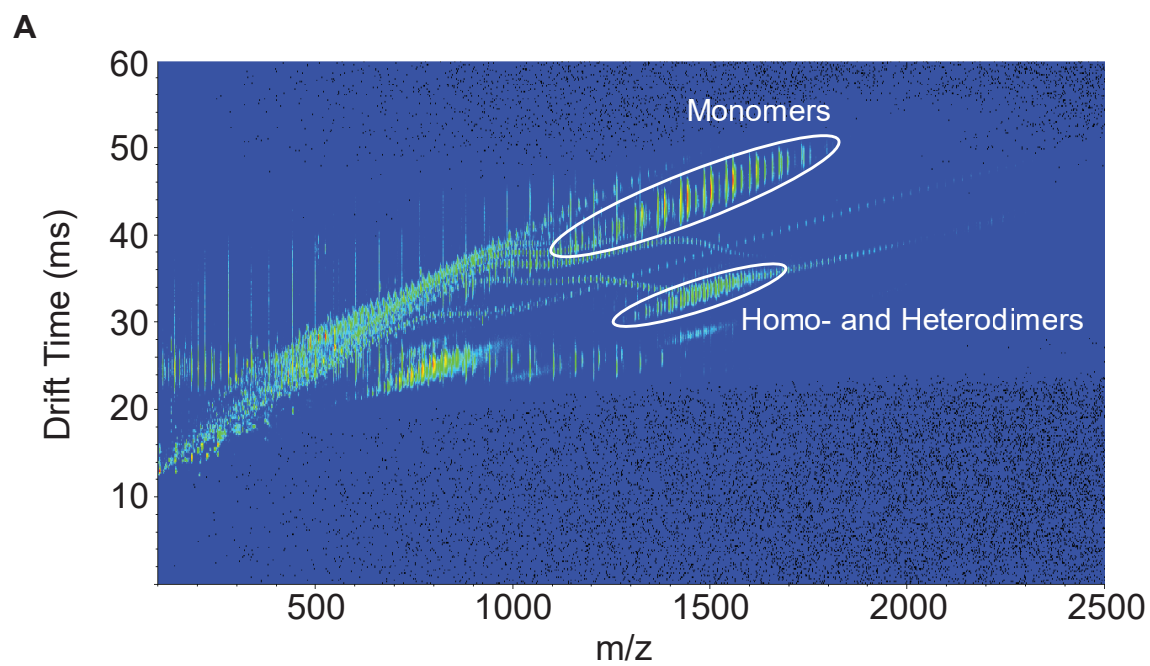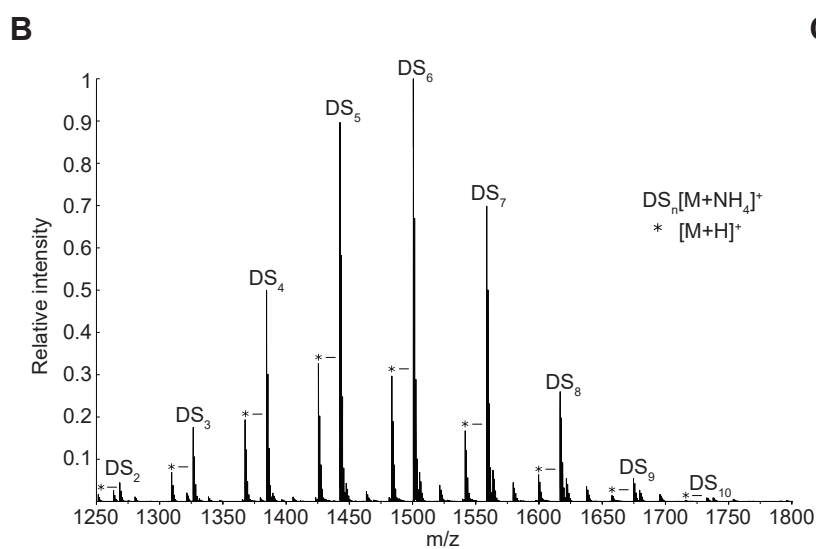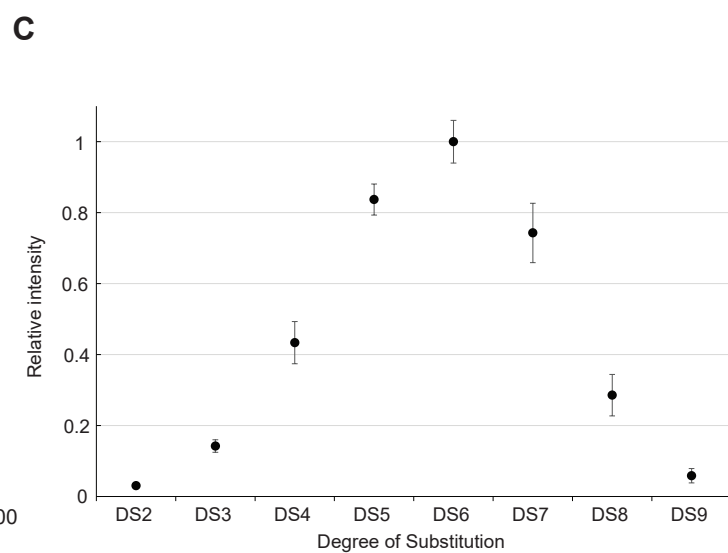

Supplement: Supplementary file 9 — Figure S9. The 2‐hydroxypropyl‐β‐cyclodextrin (HP‐β‐CD) mixture. Adjustment of the P‐value for multiple tests was performed using Benjamini‐Hochberg. *P < 0.05, **P < 0.01, ***P < 0.001, ****P < 0.0001; ns, not significant. [file MDS-37-80-s001.pdf]
